# Genomic Insights into Virulence Factors and Multi-Drug Resistance in *Clostridium perfringens* IRMC2505A

**DOI:** 10.3390/toxins15060359

**Published:** 2023-05-25

**Authors:** Reem AlJindan, Doaa M. AlEraky, Maha Farhat, Noor B. Almandil, Sayed AbdulAzeez, Jesu Francis Borgio

**Affiliations:** 1 Department of Microbiology, College of Medicine, Imam Abdulrahman Bin Faisal University, Dammam 31441, Saudi Arabia; raljindan@iau.edu.sa; 2 Department of Biomedical Dental Science, Microbiology and Immunology Division, Collage of Dentistry, Dammam 31441, Saudi Arabia; dmaleraky@iau.edu.sa; 3 Department of Biochemistry, College of Medicine, Imam Abdulrahman Bin Faisal University, Dammam 31441, Saudi Arabia; mfarhat@iau.edu.sa; 4 Department of Clinical Pharmacy Research, Institute for Research and Medical Consultations (IRMC), Imam Abdulrahman Bin Faisal University, Dammam 31441, Saudi Arabia; 5 Department of Genetic Research, Institute for Research and Medical Consultations (IRMC), Imam Abdulrahman Bin Faisal University, Dammam 31441, Saudi Arabia; asayed@iau.edu.sa (S.A.); fbalexander@iau.edu.sa (J.F.B.)

**Keywords:** *Clostridium*, anaerobic pathogen, multi-drug resistant, genome mapping, resistance genes, virulence

## Abstract

*Clostridium perfringens* is a spore-forming, Gram-positive anaerobic pathogen that causes several disorders in humans and animals. A multidrug-resistant *Clostridium* strain was isolated from the fecal sample of a patient who was clinically suspected of gastrointestinal infection and had a recent history of antibiotic exposure and diarrhea. The strain was identified by 16s rRNA sequencing as *Clostridium perfringens*. The strain’s pathogenesis was analyzed through its complete genome, specifically antimicrobial resistance-related genes. The *Clostridium perfringens* IRMC2505A genome contains 19 (*Alr*, *Ddl*, *dxr*, *EF-G*, *EF-Tu*, *folA*, *Dfr*, *folP*, *gyrA*, *gyrB*, *Iso-tRNA*, *kasA*, *MurA*, *rho*, *rpoB*, *rpoC*, *S10p*, and *S12p*) antibiotic-susceptible genetic species according to the k-mer-based detection of antimicrobial resistance genes. Genome mapping using CARD and VFDB databases revealed significant (*p*-value = 1 × 10^−26^) genes with aligned reads against antibiotic-resistant genes or virulence factors, including phospholipase C, perfringolysin O, collagenase, hyaluronidase, alpha-clostripain, exo-alpha-sialidase, and sialidase activity. In conclusion, this is the first report on *C. perfringens* from Saudi Arabia that conducted whole genome sequencing of IRMC2505A and confirmed the strain as an MDR bacterium with several virulence factors. Developing control strategies requires a detailed understanding of the epidemiology of *C. perfringens*, its virulence factors, and regional antimicrobial resistance patterns.

## 1. Introduction

*Clostridium perfringens* (*C. perfringens*) is a spore-forming, Gram-positive bacterium. *C. perfringens* was first discovered in 1981 at the Johns Hopkins Hospital. It is a causative agent of gastroenteritis, myonecrosis, and enterotoxemia. Initially, it was known as *Bacillus aerogenes capsulatus*. Before the final name, *Clostridium perfringens*, this pathogen was called *Bacillus welchii* [1,2,3,4,5,6,7,8,9,10]. *C. perfringens* can produce spores that are tolerant to environmental stress and have a wide variety of toxins involved in the pathogenesis of diseases. Spores of this pathogen can tolerate cooking temperatures and survive. As a result, *C. perfringens* can grow in foods that have been stored incorrectly. The most frequent causes of outbreaks are associated with incorrectly prepared or reheated food materials such as meats, poultry, or gravy and improper management of patients with *C. perfringens* infections before the exact diagnosis of causative agents [10]. There is evidence that type A and type C toxins can harm humans. Most cases of non-foodborne diarrheal illness and food poisoning linked to *C. perfringens* are caused by type A toxin. The pathophysiology of this pathogen is mostly associated with tissue necrosis caused by the toxin of *C. perfringens*. The majority of toxins from *C. perfringens* have pore-forming behaviors that open up, allowing water to flood in and cause swelling followed by cell death. The fermentation of glucose in this pathogen results in the generation of histotoxic gas, which is a defining feature of *C. perfringens* infection [2,5,10]. This anaerobic pathogen inhabits diverse environments and causes several disorders in humans and animals, including histotoxic (gas gangrene) infections and intestinal-associated disorders. Its pathogenicity depends on its massive production of more than 20 toxins, which varies according to different strains of *C. perfringens* [1,2,3]. These strains are classified as A–G toxinotypes according to major extracellular toxins (α, β, ε, and ι toxins). Previous comparative genomic studies on this gut pathogen indicated considerable genome variation [4]. The pathogenesis of intestinal-related diseases is characterized by rapid anaerobic growth in host tissues accompanied by the in vivo production of several key pore-forming toxins, including α-, β-, and β2-toxins, which disrupt epithelial barrier function and induce tissue necrosis or histotoxic infection [4].

Phospholipase C (plc) is the main virulent factor in *C. perfringens*-associated diseases. It is a chromosomally encoded α-toxin produced by all strains of this pathogen [5]. *C. perfringens* enterotoxin (*cpe*) is a clinically important enterotoxin gene in a few strains. This enterotoxin is the etiology of most gastrointestinal-associated diseases, including antibiotic-associated diarrhea, food-poisoning outbreaks, and nosocomial diarrheal disease. Outbreaks caused by *C. perfringens* are common, with severe illness or death reported. Current public health interventions reduce contamination at the food production source and cross-contamination in the food preparation environment [6,7,8]. Different literature has reported the characteristic resistance of *C. difficile* and its genotype profile using the resolution tool provided by whole genome sequencing (WGS) to analyze genetic diversity and assess *C. difficile* strains associated with infection recurrences and hospital outbreaks. Moreover, novel strains can spread quickly across continents and nations, negatively affecting global health. Some strains are more contagious than others for various reasons, including sporulation, antibiotic resistance, and toxin production. Using a unified typing strategy such as WGS will dramatically change our understanding of epidemiology [9,10]. However, despite *C. perfringens’* worldwide distribution, limited information is available on the genome of this pathogen, particularly in Saudi Arabia. Genes associated with antimicrobial resistance in the genome of the clinical isolate *C. perfringens* IRMC2505A could account for multidrug resistance. We used the genome analysis of this clinical isolate to test this hypothesis and investigate antimicrobial resistance-related genes involved in different resistance mechanisms. The genome of *C. perfringens* IRMC2505A was completely sequenced and analyzed using bioinformatics.

## 2. Results

**Figure 1:**
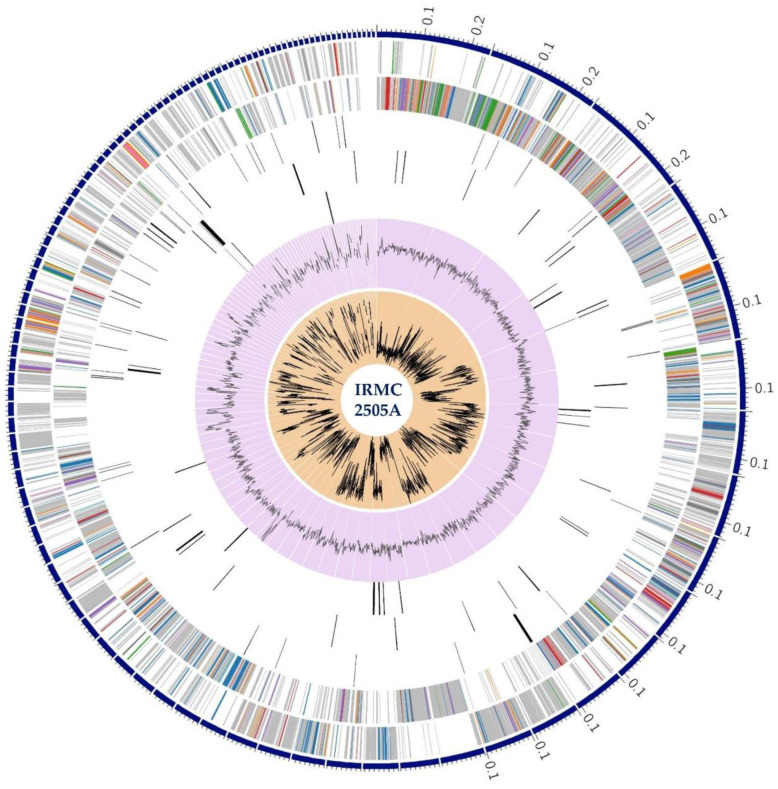
A circular graphic of the distribution of genome annotations in IRMC2505A. From the outer to inner rings, this includes the contigs, CDS on the forward strand, CDS on the reverse strand, RNA genes, CDS with homology to known antimicrobial resistance genes, CDS with homology to known virulence factors, GC content, and GC skew. The colors of the CDS on the forward and reverse strands indicate the subsystem these genes belong to (see subsystems below).

**Figure 2:**
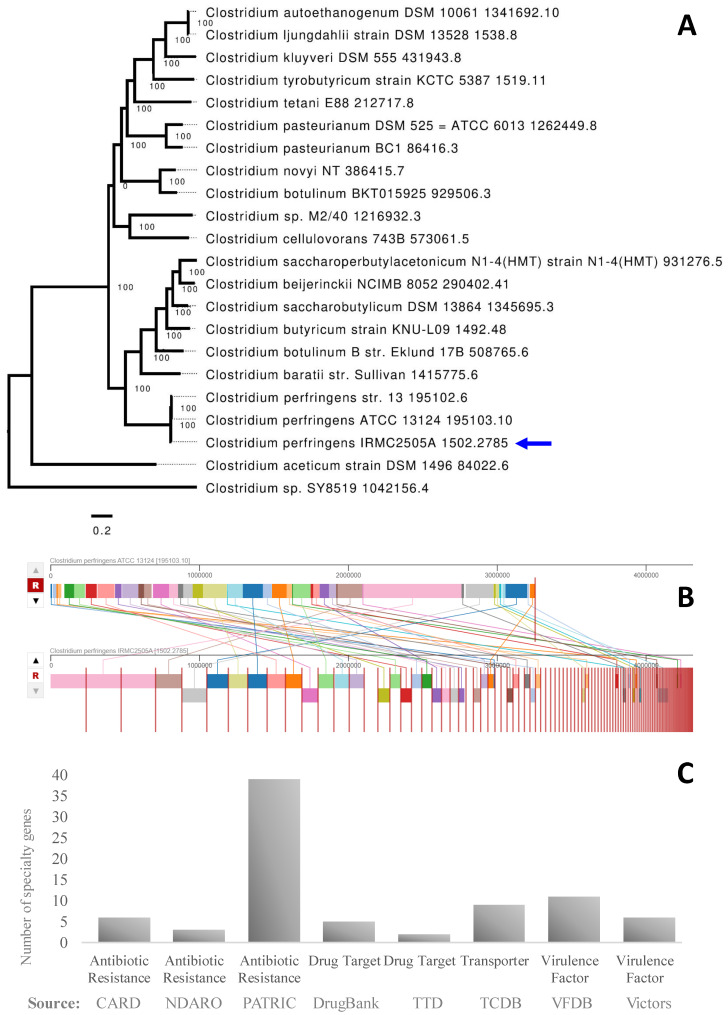
(**A**): Phylogenetic tree of the IRMC2505A genome. The blue arrow indicates the IRMC2505A genome. Tree Analysis Statistics: Requested genomes—22; Genomes with data—22; Max allowed deletions—0; Max allowed duplications—0; Single-copy genes requested—100; Single-copy genes found—100; Num protein alignments—100; Alignment program—mafft; Protein alignment time—189.9 s; Num aligned amino acids—46380; Num CDS alignments—100; Num aligned nucleotides—139140; Best protein model found by RAxML—LG; Branch support method—RAxML Fast Bootstrapping; RAxML likelihood—2571915.6763; RAxML version—8.2.11; RAxML time—3978.6 s. A list of genes used for tree analysis statistics is listed in Supplementary Table S4. (**B**): Genome comparison represents homologous regions between *Clostridium perfringens* IRMC2505A and ATCC 13124 195103.10. Alignable sequence blocks are common between the present study’s clinical isolate (lower) and the reference (upper) *C. perfringens* ATCC 13124 195103.10. Homologous locally collinear blocks between IRMC2505A and ATCC 13124 195103.10 are identified by the same color and connected with a line. (**C**): Number of specialty genes and the specific source database where homology was found.

The assembled genome of IRMC2505A had 108 contigs with a total length of 4,322,225 bp and an average G + C content of 28.28% (Table S1). This genome belongs to the Bacteria superkingdom, and its taxonomy is cellular organisms > Bacteria > Terrabacteria group > Firmicutes > Clostridia > Eubacterial > Clostridiaceae > *Clostridium* > *Clostridium perfringens.* The *Clostridium perfringens* genome has 4159 protein-coding sequences (CDS), 80 transfer RNA (tRNA) genes, and 7 ribosomal RNA (rRNA) genes (Table S1). Figure 1 shows a circular graphic of the distribution of *Clostridium perfringens* IRMC2505A genome annotations. It also presents an overview of the genome’s specific biological processes and structural complexes. The IRMC2505A genome is close to *Clostridium perfringens* based on type (Table 1 and Table 2), functional categorization (Figure 1 and Figure S1), and the number of specialty genes. We also performed a phylogenetic tree and genome comparison between *Clostridium perfringens* IRMC2505A and *Clostridium perfringens* ATCC 13124 195103.10 (Figure 2 and Figure S2). However, the genome comparison between IRMC2505A and ATCC 13124 195103.10 revealed dissimilar arrangements (Figure 2B). Protein analysis revealed the presence of 2748 proteins with functional assignments and 1411 hypothetical proteins (Table S1). The proteins with functional assignments included 870 proteins with EC numbers and 692 proteins with GO assignments. The *Clostridium perfringens* IRMC2505A genome has 3608 genus-specific protein families (PLFams) and 3702 cross-genus protein families (PGFams).

### Antimicrobial Resistance Genes

**Figure 3:**
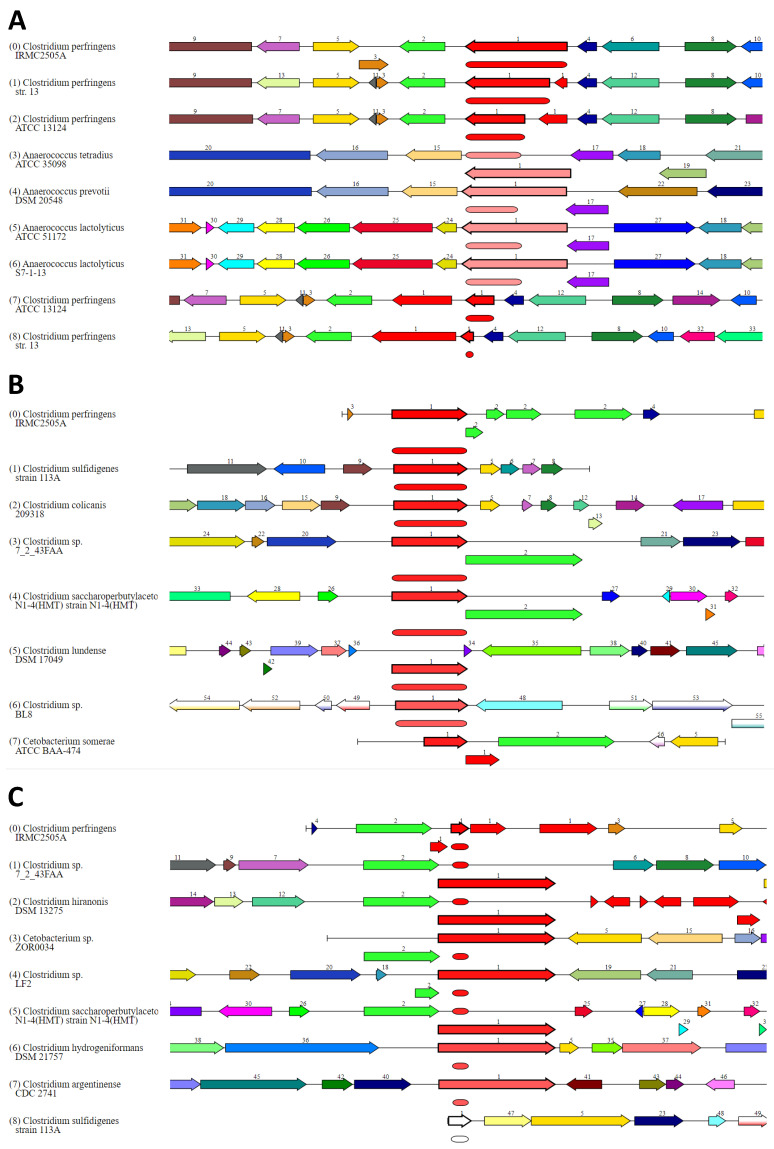
Regional view comparison of resistant genes identified in the IRMC2505A genome using the PATRIC cross-genus families (PGfams) method with reference genomes in the 10,000 bp region around the selected gene. Label 1 indicates the gene of interest. The forward and reverse orientations of genes in regions around the selected gene are indicated by arrows. Genes displaying identical colors indicate similar homology. (**A**): putative membrane protein (Classification: antibiotic target modifying enzyme, peptide antibiotic resistance gene), E-value = 0. (**B**): tetracycline-resistant MFS efflux pump ≥ TetA(P) (classification: efflux pump conferring antibiotic resistance), E-value: 1 × 10^−234^. (**C**): Tetracycline-resistant ribosomal protection type ≥ TetB(P) (classification: antibiotic target protection protein, tetracycline-resistant gene), E-value: 5 × 10^−40^.

The CARD (comprehensive antibiotic resistance database), NDARO (national database of antibiotic-resistant organisms), and PATRIC (Pathosystems Resource Integration Center) databases revealed antimicrobial resistance genes (Table 1 and Table 2; Supplementary Tables S2 and S3). Nineteen (*Alr*, *Ddl*, *dxr*, *EF-G*, *EF-Tu*, *folA*, *Dfr*, *folP*, *gyrA*, *gyrB*, *Iso-tRNA*, *kasA*, *MurA*, *rho*, *rpoB*, *rpoC*, *S10p*, and *S12p*) antibiotic targets in susceptible species were identified in the genome of *Clostridium perfringens* IRMC2505A using k-mer-based detection methods for AMR genes (Table 1). Three genes (*GdpD*, *MprF*, and *PgsA*) involved in protein-altering cell wall charge conferring antibiotic resistance were also identified in the IRMC2505A genome. Only one gene in each category was associated with the resistant mechanism of the isolate *Clostridium perfringens:* antibiotic inactivation enzyme (*NimB*), antibiotic target protection protein (*TetB*(*P*)), antibiotic target replacement protein (*fabV*), efflux pump conferring antibiotic resistance (*TetA*(*P*)), gene conferring resistance via absence (*gidB*), and protein involved in antibiotic sequestration (*FabK-like*). The putative membrane protein (*mprF*) is similar to the genome of the comprehensive antibiotic resistance database (E-value = 0.0). Tetracycline-resistant MFS efflux pump (*TetA*(*P*)) is similar to the genome reported in the NDARO, CARD, and PATRIC databases (E-value = 1 × 10^−234^) (Figure 3). We compared regional views of the putative membrane protein, tetracycline-resistant MFS efflux pump, and tetracycline-resistant ribosomal protection type genes in the IRMC2505A genome using the PATRIC cross-genus families (PGfams) method with reference genomes confirming similar subject coverage, query coverage, and identity (Table 2 and Figure 3). Seventeen virulence factors were identified in the *Clostridium perfringens* IRMC2505A genome (Table 3). More than 50% of the virulence factors are exoenzymes. Using the CARD and VFDB databases, metagenomic read mapping against the template genome revealed significant (*p*-value = 1 × 10^−26^) genes with aligned reads against antibiotic-resistant genes or virulence factors, including phospholipase C, perfringolysin O, collagenase, hyaluronidase, alpha-clostripain, exo-alpha-sialidase, and sialidase activity (Table 4).

## 3. Discussion

Although many food-borne diseases are surveyed and controlled, and several enteric organisms are monitored through databases, *Clostridium perfingens* remains one of the only toxicogenic clostridial species identified by local investigations after an outbreak occurs. The bacterium is reported worldwide and has been listed among the leading species of foodborne and non-foodborne gastroenteritis [11]. Although a serious threat is imposed by the rise of multi-drug resistant (MDR)/toxigenic strains, little is known about the prevalence and genetic composition of this pathogen in the Middle East. To our knowledge, the few studies conducted in Saudi Arabia are limited to livestock and raw animal meats. None of them involve whole genome sequencing for species identification or studying antimicrobial resistance and toxic behavioral patterns in clostridial isolates from humans [12,13].

This genomic study of *C. perfingens* was isolated from a patient in Saudi Arabia’s Eastern Province. It would be beneficial to start a surveillance program and elucidate changes in the pathogen’s genome. This information is clinically significant since the antimicrobial resistance profile and genome characteristics of these isolates are still understudied in Saudi Arabia. Additionally, recent studies have identified hypervirulent clostridial spores in new environments and genomically linked them to new matrices other than hospital environments due to uncontrolled antimicrobial usage [14,15]. Our findings may also provide insight into the adaptation of these bacteria to new niches.

We believe that a better understanding of *C. perfingens* resistance mechanisms could decrease the misuse of broad-spectrum antibiotics and AMR genes’ potential transfer to other pathogens.

### 3.1. Phylogenetic Analyses

Phylogenetic analysis confirmed the phylotype and found 100% similarity between our isolate sequences, *C. perfingens* ATCC 13124 195103.10, and *C. perfringens* str. 13 195102.6 species. The last species, *C. perfringens* str. 13, was sequenced for the first time in 2002 by Shimizu et al. It is a natural isolate found in the soil and classified as a type A strain [16]. Genome annotation distribution in IRMC2505A showed that the average genome length, GC content, and rRNA copy number were 4,322,225 bp, 28.28%, and 7, respectively. This anaerobic organism had several genes belonging to metabolism (523), protein processing (238), virulence (100), cellular processes (179), energy (135), and DNA and RNA processing (107,61). A recent study claimed that *C. perfringens* has the highest genome plasticity (by calculating the openness index (OI)) out of 51 other bacterial species. In other words, this bacterium has a highly open genome to acquire new adaptive genes for survival in hostile external environments. These properties are reflected by its low GC content (around 28%) and high 16S rRNA gene copy number [17].

### 3.2. Antimicrobial Resistance

Many research studies have indicated the emergence of multi-resistant strains of clostridial species and their significant risk to humans. In this study, the antimicrobial resistance profile of *C. perfingens* showed a multi-drug-resistant isolate involving nineteen resistance genes with different resistance mechanisms. Most of these genes were in the “antibiotic target in susceptible species” category. Many of these identical genes, with their antimicrobial resistance mechanisms, have been identified and described in different gram-negative bacteria where homologous combinations are found [18].

Predicting AMR genes based on NDARO/CARD/PATRIC databases showed tetracycline resistance (% query coverage and % identity > 97%) and defensin-like cationic antimicrobial peptides (CAMPs) (% query coverage = 100% and % identity = 95%). We detected two tetracycline-resistant genes, *tetA*(*P*) and *tetB*(*P*), which are known to encode proteins that mediate active tetracycline efflux and ribosomal protection tetracycline-resistant mechanisms, respectively [19]. The anti-defensins gene (*mprF*) reported codes for multiple peptide resistance factor proteins, which are involved in CAMP resistance [20]. Moreover, elfamycin-resistant genes were also detected with high query coverage (96%) but displayed an identity of 83%. These proteins are known as inhibitors of the Elongation Factor-Thermo Unstable (EF-Tu) used in the cell to translate mRNA transcripts into proteins [21].

Antimicrobial agents are widely used in animals as growth promoters and can lead to the emergence of antimicrobial-resistant enteric microflora, including *C. perfringens* and its spores [22]. Hence, antibiotic-resistant *C. perfringens* should be investigated in many matrixes (animal, human, food, soil…) [23]. Motamedi et al. conducted the first systematic review in 2021 on *C. perfringens* as a causative agent of antibiotic-associated diarrhea (AAD) in hospitalized patients [24]. These studies were conducted mainly in Asia, Europe, and Africa and showed 14.9% *C. perfringens* among patients. However, insufficient data were found in these areas. Consequently, this finding stresses the urgent need for a surveillance program to control AAD and guarantee efficient treatment.

A similar situation was observed in Saudi Arabia, where a recent meta-analysis (2022) on the frequency of antibiotic-resistant *Clostridium* species found no evidence for the antimicrobial resistance patterns reported in KSA. Most of these limited studies used conventional methods (selective culture on agar media, E-tests, and disk diffusion assays) [25]. These reference methods only isolate and identify the studied bacteria and do not distinguish between different pathogens based on their virulence phenotypes. Limited research studies in KSA investigated the resistance and occurrence of *C. perfringens* in camel meat. They showed these bacteria’s high resistance to tetracycline and ceftiofur drugs and, to a lesser extent, other antimicrobial agents (erythromycin, oxytetracycline, penicillin, metronidazole, clindamycin, and lincomycin) [12,26,27].

### 3.3. Toxigenicity

*C. perfringens* is also known for its toxigenicity. Five toxinotypes (A–G) were historically identified based on the toxin produced by the strain (alpha, beta, epsilon, and iota toxins). Afterward, two new toxinotypes (F and G) were added to the scheme, introducing enterotoxin CPE (but not CPB, ETX, or ITX) and NetB toxin, respectively [28]. Many virulence factors contribute to this pathogen’s toxicity. *C. perfringens* is known to produce a wide range of degradative enzymes, including hydrolytic enzymes, membrane-damaging enzymes, pore-forming toxins, and intracellular toxins (proteases, hyaluronidase, collagenase, endoglycosidases, etc.). The virulence analysis of our bacterial isolate showed a variable array of virulence genes and a 97% similarity to the *C. perfringens* genome. High query coverage sequences (≥97%) were distributed as follows: Sequences correlated to phospholipase C (*plc*) encoding the alpha toxin, sialidase genes (*nanH*, *nanI*), alpha-clostripain genes (*cloSI*), hyaluronidase genes (*nagJ*, *nagH*), and collagenase genes (*colA*) encoding the κ-toxin. These genes are chromosomal and located specifically in the conserved region of the *C. perfringens* chromosome [29]. There has been no WGS study on *C. perfringens* in Saudi Arabia, including the Arabian Peninsula [30].

In conclusion, this WGS study of *C. perfringens* showed an MDR bacterium with several virulence factors. To our knowledge, our WGS study is one of the first studies conducted in Saudi Arabia and the Arabian Peninsula on *C. perfringens*, where national databases are still inexistent. To guarantee a complete understanding of *C. perfringens’* epidemiology, a full study of this pathogen’s virulence factors, and antimicrobial resistance patterns is essential. Future studies will also be beneficial in developing an effective disease control strategy.

## 4. Materials and Methods

### 4.1. Isolation and DNA Extraction of C. perfringens IRMC2505A

This project was submitted for ethical approval to the ethical committee of Imam Abdulrahman Bin Faisal University (IRB-2022-01-398). All procedures were performed in accordance with the 1964 Helsinki Declaration and its later amendments or comparable ethical standards.

*A Clostridium perfringens* strain was isolated using cycloserine, cefoxitin, and fructose agar medium from the fecal sample of a patient who was clinically suspected of gastrointestinal infection and had a recent history of antibiotic exposure and diarrhea. A positive sample, tested for both toxins A and B by GeneXpert, was cultured on cycloserine cefoxitin fructose agar selective medium (CCFA) (MOLEQULE-ON, Auckland, New Zealand) under anaerobic conditions at 37 °C for 24 h. The total DNA was extracted using the Gentra Puregene Yeast/Bact. Kit (Qiagen, Hilden, Germany). The purity, quality, and quantity of genomic DNA were measured using Nanodrop 2000 (Thermo Scientific, Waltham, MA, USA) as described in the manufacturer’s instructions. The isolate was PCR amplified, and *16S rRNA* was sequenced to confirm the isolate.

### 4.2. Virulence Genes Identification Using Whole Genome Sequencing

The whole genome of isolate IRMC2505A was sequenced using the Illumina platform. The paired reads were assembled and annotated using PATRIC (BV-BRC 3.28.5) [31] and the RAST tool kit (RASTtk 1.3.0) [32]. We calculated the number of contigs and average G + C content and identified the taxonomy of the genome. We also predicted the proteins and their functional assignments with the Enzyme Commission (EC) [33], Gene Ontology (GO) [34], pathways [35], protein family types [36], and subsystems of protein complexes [37]. Specialty genes in the genome of IRMC2505A were identified using specific source databases for known transporters [38], virulence factors [39,40], drug targets [41,42], and antibiotic-resistant genes [43]. We detected k-mer-based antimicrobial resistance (AMR) genes [31]. We phylogenetically analyzed the IRMC2505A genome using 100 genes from the National Center for Biotechnology Information (NCBI) reference and representative genomes by Mash/MinHash (Mash v2.3) and PGFams (PATRIC, BV-BRC 3.28.5) [36] aligned with MUSCLE [44], a matrix analysis using RaxML (version 8) with fast bootstrapping [45,46]. Metagenomic read mapping against the selected template was analyzed using the CARD (2020) and VFDB (2019) databases through k-mer alignment [47].

## Figures and Tables

**Table 1:** Mechanisms of antimicrobial-resistant genes identified in the *Clostridium perfringens* IRMC2505A genome.

S. No.	Antimicrobial Resistant Mechanism	Genes
1	Antibiotic inactivation enzyme	*NimB*
2	Antibiotic target in susceptible species	*Alr*, *Ddl*, *dxr*, *EF-G*, *EF-Tu*, *folA*, *Dfr*, *folP*, *gyrA*, *gyrB*, *Iso-tRNA*, *kasA*, *MurA*, *rho*, *rpoB*, *rpoC*, *S10p*, *S12p*
3	Antibiotic target protection protein	*TetB(P)*
4	Antibiotic target replacement protein	*FabV*
5	Efflux pump conferring antibiotic resistance	*TetA(P)*
6	Gene conferring resistance via absence	*GidB*
7	Protein altering cell wall charge conferring antibiotic resistance	*GdpD*, *MprF*, *PgsA*
8	Protein involved in antibiotic sequestration	*FabK-like*

**Table 2:** List of antimicrobial resistance genes identified in the *Clostridium perfringens* IRMC2505A genome.

	Source	Gene	Product/Function	Classification	Subject Coverage	Query Coverage	Identity	E-Value
1	NDARO/CARD	*tetB(P)*	Tetracycline resistance, ribosomal protection type ≥ TetB(P)	antibiotic target protection protein, tetracycline-resistant gene	13	91	95	5 × 10^−40^
2	CARD/NDARO	*tetB(P)*	Tetracycline resistance, ribosomal protection type ≥ TetB(P)	antibiotic target protection protein, tetracycline-resistant gene	48	99	98	1 × 10^−180^
3	CARD/PATRIC	*EF-Tu*	Translation elongation factor Tu	antibiotic resistant gene variant or mutant, elfamycin-resistant gene/antibiotic target in susceptible species	6	96	83	7 × 10^−3^
4	NDARO/CARD/PATRIC	*tetA(P)*	Tetracycline resistance, MFS efflux pump ≥ TetA(P)	efflux pump conferring antibiotic resistance	100	100	98	1 × 10^−234^
5	CARD	*mprF*	putative membrane protein	antibiotic target modifying enzyme, peptide antibiotic resistance gene	100	100	95	0.0

CARD: Comprehensive Antibiotic Resistance Database; NDARO: National Database of Antibiotic Resistant Organisms; PATRIC: Pathosystems Resource Integration Center. Complete list of antimicrobial resistance genes identified in the *Clostridium perfringens* IRMC2505A genome with classifications is listed in Supplementary Tables S2 and S3.

**Table 3:** List of virulence factors identified in the *Clostridium perfringens* IRMC2505A genome.

Source	Source ID	Source Organism	Gene	Product	Classification	Subject Coverage	Query Coverage	Identity	E-Value
Victors	15676067	*Neisseria meningitidis* MC58	*tufA*	Translation elongation factor Tu		5	92	86	3 × 10^−3^
VFDB	VFG002285	*Clostridium perfringens* ATCC 13124	*nanH*	Sialidase (EC 3.2.1.18)	Exoenzyme, Carbohydrate-active enzyme, Sialidase	100	100	98	1 × 10^−226^
Victors	110801372	*Clostridium perfringens* ATCC 13124	*virS*	Hypothetical protein		99	100	95	1 × 10^−243^
Victors	18309145	*Clostridium perfringens* str. 13	*pfoA*	Thiol-activated cytolysin		92	88	87	1 × 10^−243^
Victors	15676067	*Neisseria meningitidis* MC58	*tufA*	Translation elongation factor Tu		5	92	86	3 × 10^−3^
VFDB	VFG002278	*Clostridium perfringens* str. 13	*nagI*	Protein O-GlcNAcase (EC 3.2.1.169)	Exoenzyme, Carbohydrate-active enzyme, Hyaluronidase	100	100	96	0.0
Victors	29376182	*Enterococcus faecalis* V583	*EF1623*	Ethanolamine utilization protein similar to PduA/PduJ		91	91	92	4 × 10^−38^
VFDB	VFG002274	*Clostridium perfringens* str. 13	*plc*	Broad-substrate range phospholipase C (EC 3.1.4.3)	Toxin, Zinc-metallophospholipase C	100	100	99	1 × 10^−242^
VFDB	VFG002282	*Clostridium perfringens* str. 13	*cloSI*	Hypothetical protein	Exoenzyme, Cysteine endopeptidase	99	100	97	1 × 10^−306^
VFDB	VFG002279	*Clostridium perfringens* str. 13	*nagJ*	Protein O-GlcNAcase (EC 3.2.1.169)	Exoenzyme, Carbohydrate-active enzyme, Hyaluronidase	100	100	98	0.0
Victors	18309018	*Clostridium perfringens* str. 13	*plc*	Broad-substrate range phospholipase C (EC 3.1.4.3)		100	100	99	1 × 10^−242^
VFDB	VFG002281	*Clostridium perfringens* str. 13	*nagL*	Hypothetical protein	Exoenzyme, Carbohydrate-active enzyme, Hyaluronidase	100	100	95	0.0
VFDB	VFG002283	*Clostridium perfringens* str. 13	*nanI*	Sialidase (EC 3.2.1.18)	Exoenzyme, Carbohydrate-active enzyme, Sialidase	100	100	99	0.0
VFDB	VFG002276	*Clostridium perfringens* str. 13	*colA*	Microbial collagenase (EC 3.4.24.3)	Exoenzyme, Collagenase	100	100	98	0.0
VFDB	VFG002284	*Clostridium perfringens* str. 13	*nanJ*	Sialidase (EC 3.2.1.18)	Exoenzyme, Carbohydrate-active enzyme, Sialidase	100	100	95	0.0
VFDB	VFG002275	*Clostridium perfringens* str. 13	*pfoA*	Thiol-activated cytolysin	Toxin, Membrane-damaging, Pore-forming, Channel-forming involving beta-sheet-containing toxin (beta-barrel), Cholesterol-dependent cytolysin	92	88	87	1 × 10^−243^
VFDB	VFG002277	*Clostridium perfringens* str. 13	*nagH*	Hyaluronoglucosaminidase (EC 3.2.1.35)	Exoenzyme, Carbohydrate-active enzyme, Hyaluronidase	100	100	97	0.0

**Table 4:** Metagenomic read mapping of *Clostridium perfringens* IRMC2505A against the templates using the CARD and VFDB databases.

Template	Gene Function	Genome	Score	Expected	Template Length	Template Identity	Template Coverage	Query Identity	Query Coverage	Depth	q-Value	*p*-Value
CARD|ABG86067.1	*Clostridium perfringens* mprF	$	1395	1	1710	93.8	100	93.8	100	1	1390.34	1.00 × 10^−26^
CARD|AAA20116.1	tetA(P)	#	1218	1	1263	98.73	100	98.73	100	1	1214.3	1.00 × 10^−26^
VFDB|VFG002274	phospholipase C	*	1164	7	1197	99	100	99	100	1	1140.8	1.00 × 10^−26^
VFDB|VFG002275	perfringolysin O	*	915	9	1503	86.56	98.27	88.08	101.76	0.98	885.75	1.00 × 10^−26^
VFDB|VFG002276	collagenase	*	3177	19	3315	98.58	100	98.58	100	1	3117.65	1.00 × 10^−26^
VFDB|VFG002277	hyaluronidase	*	4467	27	4887	97.11	100	97.11	100	1	4384.16	1.00 × 10^−26^
VFDB|VFG002278	hyaluronidase	*	3537	23	3894	96.92	100	96.92	100	1	3468.34	1.00 × 10^−26^
VFDB|VFG002279	hyaluronidase	*	2769	18	3006	97.34	100	97.34	100	1	2714.3	1.00 × 10^−26^
VFDB|VFG002281	hyaluronidase	*	2976	20	3384	95.95	100	95.95	100	1	2914.96	1.00 × 10^−26^
VFDB|VFG002282	alpha-clostripain	*	1459	10	1575	97.65	100	97.65	100	1	1428.83	1.00 × 10^−26^
VFDB|VFG002283	exo-alpha-sialidase	*	1995	13	2085	98.51	100	98.51	100	1	1955.87	1.00 × 10^−26^
VFDB|VFG002284	exo-alpha-sialidase	*	3206	21	3522	97.02	100	97.02	100	1	3143.05	1.00 × 10^−26^
VFDB|VFG002285	sialidase	‡	1026	7	1149	96.34	100	96.34	100	1	1003.63	1.00 × 10^−26^

Template: reference gene sequence; Genome: Genome that contains template gene; $: *Clostridium perfringens* SM101; #: *Clostridium perfringens*; *: *Clostridium perfringens* str. 13; ‡: *Clostridium perfringens* ATCC 13124. Score: global alignment score of the template; Expected: expected alignment score; Template length: Template gene length in nucleotides; Template Identity: Percentage (%) of identity between the query and template over the length of the matching query; template Coverage: Percentage (%) of the template that is covered by the query; Query Identity: Percentage (%) of identity between the query and template sequence over the length of the matching query sequence; Query Coverage: length of the matching query divided by the template length; Depth: Number of times the template is covered by the query; q-value: Quantile from McNemar’s test to evaluate whether the current template is significant; *p*-value: *p*-value corresponding to the obtained q-value.

## Data Availability

All data will be available on reasonable request from the corresponding author.

## Supplementary Materials

The following supporting information can be downloaded at: https://www.mdpi.com/article/10.3390/toxins15060359/s1, Figure S1: A: A circular graphical display of the distribution of the genome annotations in IRMC2505A. B: An overview of the subsystems of genes for the genome IRMC2505A; Figure S2: Proteome comparison. List of tracks, from outside to inside: *Clostridium perfringens* ATCC 13124 (195103.10) and *Clostridium perfringens* IRMC2505A (1502.2785); Table S1: Assembly Details and annotated features of *Clostridium perfringens* IRMC2505A; Table S2: List of antimicrobial resistance genes identified in the genome of *Clostridium perfringens* IRMC2505A; Table S3: Functions and classifications of list of antimicrobial resistance genes in the genome of *C. perfringens* IRMC2505A; Table S4: Gene families are ranked by alignment score for phylogenetic tree of IRMC2505A genome.
