# Supplementary material for: Genomic Insights into Virulence Factors and Multi-Drug Resistance in Clostridium perfringens IRMC2505A
Source: Toxins (Basel). 2023 May 25;15(6):359. doi: 10.3390/toxins15060359 (PMC10302382; doi:10.3390/toxins15060359)
Supplement: Supplementary file 1 [file toxins-15-00359-s001.zip › toxins-2394276-supplementary.pdf]

Genomic Insights into Virulence Factors and Multi-Drug Resistance in Clostridium perfringens IRMC2505A

Supplementary Table S1: Assembly Details and annotated features of *Clostridium perfringens* IRMC2505A.

General Info	
**Genome Name**	*Clostridium perfringens* IRMC2505A
**Taxonomy Info**	
Taxon ID	1502
Superkingdom	Bacteria
Phylum	Firmicutes
Class	Clostridia
Order	Eubacteriales
Family	Clostridiaceae
Genus	*Clostridium*
Species	*Clostridium perfringens*
**Status**	
Genome Status	WGS
**Genome Statistics**	
Contigs	108
Genome Length	4322225
GC Content	28.275791
Contig L50	15
Contig N50	91957
**Annotation Statistics**	
tRNA	80
rRNA	7
CDS	4159
CDS Ratio	0.96223587
Hypothetical CDS	1411
Hypothetical CDS Ratio	0.4308728

PLFAM CDS	3608
PLFAM CDS Ratio	0.8675162
**Genome Quality**	
Coarse Consistency	99.6
Fine Consistency	94.5
CheckM Completeness	99.9
CheckM Contamination	12.3
Genome Quality	Good

Supplementary Table S2: List of antimicrobial resistance genes identified in the genome of *Clostridium perfringens* IRMC2505A.

	Source	Gene	Product/Function	Classification	Subject Coverage	Query Coverage	Identity	E-value*
1	PATRIC	*EF-G*	Translation elongation factor G	antibiotic target in susceptible species				
2	NDARO	*TetB(P)*	Tetracycline resistance, ribosomal protection type => TetB(P)		13	91	95	5e-40
3	PATRIC	*S12p*	SSU ribosomal protein S12p (S23e)	antibiotic target in susceptible species				
4	PATRIC	*EF-Tu*	Translation elongation factor Tu	antibiotic target in susceptible species				
5	PATRIC	*TetB(P)*	Tetracycline resistance, ribosomal protection type => TetB(P)	antibiotic target protection protein				
6	PATRIC	*MurA*	UDP-N-acetylglucosamine 1-carboxyvinyltransferase (EC 2.5.1.7)	antibiotic target in susceptible species				
7	PATRIC	*Alr*	Alanine racemase (EC 5.1.1.1)	antibiotic target in susceptible species				
8	PATRIC	*TetB(P)*	Tetracycline resistance, ribosomal protection type => TetB(P)	antibiotic target protection protein				
9	PATRIC	*gidB*	16S rRNA (guanine(527)-N(7))-methyltransferase (EC 2.1.1.170)	gene conferring resistance via absence				
10	PATRIC	*gyrB*	DNA gyrase subunit B (EC 5.99.1.3)	antibiotic target in susceptible species				
11	PATRIC	*Iso-tRNA*	Isoleucyl-tRNA synthetase (EC 6.1.1.5)	antibiotic target in susceptible species				
12	PATRIC	*Alr*	Alanine racemase (EC 5.1.1.1)	antibiotic target in susceptible species				
13	CARD	*EF-Tu*	Translation elongation factor Tu	antibiotic resistant gene variant or mutant,elfamycin resistance gene	6	96	83	7e-03

14	PATRIC	*EF-Tu*	Translation elongation factor Tu	antibiotic target in susceptible species				
15	NDARO	*TetA(P)*	Tetracycline resistance, MFS efflux pump => TetA(P)		100	100	100	1e-237
16	PATRIC	*FabK-like*	Putative FabK-like enoyl-[acyl-carrier-protein] reductase	protein involved in antibiotic sequestration				
17	CARD	*mprF*	putative membrane protein	antibiotic target modifying enzyme,peptide antibiotic resistance gene	100	100	95	0.0
18	PATRIC	*NimB*	Nitroimidazole resistance protein NimB	antibiotic inactivation enzyme				
19	PATRIC	*gyrB*	DNA gyrase subunit B (EC 5.99.1.3)	antibiotic target in susceptible species				
20	PATRIC	*TetB(P)*	Tetracycline resistance, ribosomal protection type => TetB(P)	antibiotic target protection protein				
21	PATRIC	*S10p*	SSU ribosomal protein S10p (S20e)	antibiotic target in susceptible species				
22	PATRIC	*GdpD*	Glycerophosphoryl diester phosphodiesterase (EC 3.1.4.46)	protein altering cell wall charge conferring antibiotic resistance				
23	PATRIC	*kasA*	3-oxoacyl-[acyl-carrier-protein] synthase, KASII (EC 2.3.1.179)	antibiotic target in susceptible species				
24	CARD	*tetA(P)*	Tetracycline resistance, MFS efflux pump => TetA(P)	efflux pump conferring antibiotic resistance	100	100	98	1e-234
25	PATRIC	*fabV*	Enoyl-[acyl-carrier-protein] reductase [NADH] (EC 1.3.1.9), FabV => refractory to triclosan	antibiotic target replacement protein				
26	PATRIC	*MurA*	UDP-N-acetylglucosamine 1-carboxyvinyltransferase (EC 2.5.1.7)	antibiotic target in susceptible species				
27	CARD	*EF-Tu*	Translation elongation factor Tu	antibiotic resistant gene variant or	6	96	83	7e-03

				mutant,elfamycin resistance gene				
28	CARD	*tetB(P)*	Tetracycline resistance, ribosomal protection type => TetB(P)	antibiotic target protection protein,tetracycline resistance gene	48	99	98	1e-180
29	PATRIC	*TetA(P)*	Tetracycline resistance, MFS efflux pump => TetA(P)	efflux pump conferring antibiotic resistance				
30	PATRIC	*dxr*	1-deoxy-D-xylulose 5-phosphate reductoisomerase (EC 1.1.1.267)	antibiotic target in susceptible species				
31	PATRIC	*MprF*	L-O-lysylphosphatidylglycerol synthase (EC 2.3.2.3)	protein altering cell wall charge conferring antibiotic resistance				
32	NDARO	*TetB(P)*	Tetracycline resistance, ribosomal protection type => TetB(P)		48	99	98	1e-180
33	PATRIC	*rho*	Transcription termination factor Rho	antibiotic target in susceptible species				
34	PATRIC	*gyrA*	DNA gyrase subunit A (EC 5.99.1.3)	antibiotic target in susceptible species				
35	PATRIC	*GdpD*	Glycerophosphoryl diester phosphodiesterase (EC 3.1.4.46)	protein altering cell wall charge conferring antibiotic resistance				
36	PATRIC	*PgsA*	CDP-diacylglycerol--glycerol-3-phosphate 3-phosphatidyltransferase (EC 2.7.8.5)	protein altering cell wall charge conferring antibiotic resistance				
37	PATRIC	*gidB*	16S rRNA (guanine(527)-N(7))-methyltransferase (EC 2.1.1.170)	gene conferring resistance via absence				
38	PATRIC	*gyrA*	DNA gyrase subunit A (EC 5.99.1.3)	antibiotic target in susceptible species				
39	PATRIC	*rpoC*	DNA-directed RNA polymerase beta' subunit (EC 2.7.7.6)	antibiotic target in susceptible species				
40	PATRIC	*TetB(P)*	Tetracycline resistance, ribosomal protection type => TetB(P)	antibiotic target protection protein				

41	PATRIC	*MurA*	UDP-N-acetylglucosamine 1-carboxyvinyltransferase (EC 2.5.1.7)	antibiotic target in susceptible species				
42	CARD	*tetB(P)*	Tetracycline resistance, ribosomal protection type => TetB(P)	antibiotic target protection protein,tetracycline resistance gene	13	91	95	5e-40
43	PATRIC	*folP*	Dihydropteroate synthase (EC 2.5.1.15)	antibiotic target in susceptible species				
44	PATRIC	*Ddl*	D-alanine--D-alanine ligase (EC 6.3.2.4)	antibiotic target in susceptible species				
45	PATRIC	*Ddl*	D-alanine--D-alanine ligase (EC 6.3.2.4)	antibiotic target in susceptible species				
46	PATRIC	*Alr*	Alanine racemase (EC 5.1.1.1)	antibiotic target in susceptible species				
47	PATRIC	*folA, Dfr*	Dihydrofolate reductase (EC 1.5.1.3)	antibiotic target in susceptible species				
48	PATRIC	*rpoB*	DNA-directed RNA polymerase beta subunit (EC 2.7.7.6)	antibiotic target in susceptible species				

*E-values were available only for CARD (Comprehensive Antibiotic Resistance Database) and NDARO (National Database of Antibiotic Resistant Organisms). PATRIC: Pathosystems Resource Integration Center.

Supplementary Table S3: Functions and classifications of list of antimicrobial resistance genes in the genome of *C. perfringens* IRMC2505A.

<table>
<tr><th>Evidence</th><th>Property</th><th>Source</th><th>Genome Name</th><th>BRC ID</th><th>RefSeq Locus Tag</th><th>Alt Locus Tag</th><th>Source ID</th><th>Source Organism</th><th>Gene</th><th>Product</th><th>Function</th><th>Classification</th><th>PubMed</th><th>Subject Coverage</th><th>Query Coverage</th><th>Identity</th><th>E-value</th></tr>
<tr><td>BLAT</td><td>Transporter</td><td>TCDB</td><td>Clostridium perfringens IRMC2505A</td><td>fig|1502.2785.peg.21</td><td></td><td></td><td>Q08637</td><td>Enterococcus hirae</td><td></td><td>V-type ATP synthase subunit B (EC 3.6.3.14)</td><td>the h(+)- or na(+)-translocating f-type, v-type and a-type atpase (f-atpase) superfamily.</td><td>3.A.2.2.2</td><td>8144530;8157629;8373385</td><td>99</td><td>99</td><td>82</td><td>1e-222</td></tr>
<tr><td>BLAT</td><td>Virulence Factor</td><td>Victors</td><td>Clostridium perfringens IRMC2505A</td><td>fig|1502.2785.peg.824</td><td></td><td></td><td>15676067</td><td>Neisseria meningitidis MC58</td><td>tufA</td><td>Translation elongation factor Tu</td><td></td><td></td><td>11062540</td><td>5</td><td>92</td><td>86</td><td>3e-03</td></tr>
<tr><td>K-mer Search</td><td>Antibiotic Resistance</td><td>PATRIC</td><td>Clostridium perfringens IRMC2505A</td><td>fig|1502.2785.peg.3739</td><td></td><td></td><td></td><td></td><td>EF-G</td><td>Translation elongation factor G</td><td>Translation elongation factor G</td><td>antibiotic target in susceptible species</td><td>17980694</td><td></td><td></td><td></td><td></td></tr>
<tr><td>BLAT</td><td>Antibiotic Resistance</td><td>NDARO</td><td>Clostridium perfringens IRMC2505A</td><td>fig|1502.2785.peg.3985</td><td></td><td></td><td>WP_012478245.1</td><td>Clostridium perfringens</td><td></td><td>Tetracycline resistance, ribosomal protection type => TetB(P)</td><td>tetracycline resistance ribosomal protection protein TetB(P)</td><td></td><td></td><td>13</td><td>91</td><td>95</td><td>5e-40</td></tr>
<tr><td>K-mer Search</td><td>Antibiotic Resistance</td><td>PATRIC</td><td>Clostridium perfringens IRMC2505A</td><td>fig|1502.2785.peg.3737</td><td></td><td></td><td></td><td></td><td>S12p</td><td>SSU ribosomal protein S12p (S23e)</td><td>SSU ribosomal protein S12p (S23e)</td><td>antibiotic target in susceptible species</td><td>7934937</td><td></td><td></td><td></td><td></td></tr>
<tr><td>K-mer Search</td><td>Antibiotic Resistance</td><td>PATRIC</td><td>Clostridium perfringens IRMC2505A</td><td>fig|1502.2785.peg.3726</td><td></td><td></td><td></td><td></td><td>EF-Tu</td><td>Translation elongation factor Tu</td><td>Translation elongation factor Tu</td><td>antibiotic target in susceptible species</td><td>364475;9678602</td><td></td><td></td><td></td><td></td></tr>
<tr><td>BLAT</td><td>Virulence Factor</td><td>VFDB</td><td>Clostridium perfringens IRMC2505A</td><td>fig|1502.2785.peg.1184</td><td></td><td></td><td>VFG002285</td><td>Clostridium perfringens ATCC 13124</td><td>nanH</td><td>Sialidase (EC 3.2.1.18)</td><td></td><td>Exoenzyme,Carbohydrate-active enzyme,Sialidase</td><td></td><td>100</td><td>100</td><td>98</td><td>1e-226</td></tr>
<tr><td>BLAT</td><td>Drug Target</td><td>TTD</td><td>Clostridium perfringens IRMC2505A</td><td>fig|1502.2785.peg.824</td><td></td><td></td><td>TTDI02058</td><td></td><td></td><td>Translation elongation factor Tu</td><td></td><td></td><td></td><td>5</td><td>88</td><td>86</td><td>3e-02</td></tr>
<tr><td>K-mer Search</td><td>Antibiotic Resistance</td><td>PATRIC</td><td>Clostridium perfringens IRMC2505A</td><td>fig|1502.2785.peg.3987</td><td></td><td></td><td></td><td></td><td>TetB(P)</td><td>Tetracycline resistance, ribosomal protection type => TetB(P)</td><td>Tetracycline resistance, ribosomal protection type => TetB(P)</td><td>antibiotic target protection protein</td><td>8170402;11717269</td><td></td><td></td><td></td><td></td></tr>
<tr><td>K-mer Search</td><td>Antibiotic Resistance</td><td>PATRIC</td><td>Clostridium perfringens IRMC2505A</td><td>fig|1502.2785.peg.624</td><td></td><td></td><td></td><td></td><td>MurA</td><td>UDP-N-acetylglucosamine 1-carboxyvinyltransferase (EC 2.5.1.7)</td><td>UDP-N-acetylglucosamine 1-carboxyvinyltransferase (EC 2.5.1.7)</td><td>antibiotic target in susceptible species</td><td>8994972</td><td></td><td></td><td></td><td></td></tr>
<tr><td>BLAT</td><td>Transporter</td><td>TCDB</td><td>Clostridium perfringens IRMC2505A</td><td>fig|1502.2785.peg.2183</td><td></td><td></td><td>P0C2E9</td><td>Clostridium perfringens</td><td></td><td>Thiol-activated cytolysin</td><td>the thiol-activated cholesterol-dependent cytolysin (cdc) family.</td><td>1.C.12.1.1</td><td>11792842;1987025; 2878682;2888650; 8955365;9182756</td><td>92</td><td>88</td><td>87</td><td>1e-243</td></tr>
<tr><td>BLAT</td><td>Drug Target</td><td>DrugBank</td><td>Clostridium perfringens IRMC2505A</td><td>fig|1502.2785.peg.4032</td><td></td><td></td><td>Q8XL08</td><td>Clostridium perfringens (strain 13 / Type A)</td><td>nagJ</td><td>Protein O-GlcNAcase (EC 3.2.1.169)</td><td>gamma-butyrolactone</td><td>Drug target</td><td></td><td>100</td><td>100</td><td>98</td><td>0.0</td></tr>
<tr><td>K-mer Search</td><td>Antibiotic Resistance</td><td>PATRIC</td><td>Clostridium perfringens IRMC2505A</td><td>fig|1502.2785.peg.1488</td><td></td><td></td><td></td><td></td><td>Alr</td><td>Alanine racemase (EC 5.1.1.1)</td><td>Alanine racemase (EC 5.1.1.1)</td><td>antibiotic target in susceptible species</td><td>19748470;24303782</td><td></td><td></td><td></td><td></td></tr>
<tr><td>K-mer Search</td><td>Antibiotic Resistance</td><td>PATRIC</td><td>Clostridium perfringens IRMC2505A</td><td>fig|1502.2785.peg.3985</td><td></td><td></td><td></td><td></td><td>TetB(P)</td><td>Tetracycline resistance, ribosomal protection type => TetB(P)</td><td>Tetracycline resistance, ribosomal protection type => TetB(P)</td><td>antibiotic target protection protein</td><td>8170402;11717269</td><td></td><td></td><td></td><td></td></tr>
<tr><td>K-mer Search</td><td>Antibiotic Resistance</td><td>PATRIC</td><td>Clostridium perfringens IRMC2505A</td><td>fig|1502.2785.peg.3358</td><td></td><td></td><td></td><td></td><td>gidB</td><td>16S rRNA (guanine(527)-N(7))-methyltransferase (EC 2.1.1.170)</td><td>16S rRNA (guanine(527)-N(7))-methyltransferase (EC 2.1.1.170)</td><td>gene conferring resistance via absence</td><td>17238915</td><td></td><td></td><td></td><td></td></tr>
<tr><td>K-mer Search</td><td>Antibiotic Resistance</td><td>PATRIC</td><td>Clostridium perfringens IRMC2505A</td><td>fig|1502.2785.peg.3344</td><td></td><td></td><td></td><td></td><td>gyrB</td><td>DNA gyrase subunit B (EC 5.99.1.3)</td><td>DNA gyrase subunit B (EC 5.99.1.3)</td><td>antibiotic target in susceptible species</td><td>21693461;22279180;9293187</td><td></td><td></td><td></td><td></td></tr>
<tr><td>BLAT</td><td>Drug Target</td><td>TTD</td><td>Clostridium perfringens IRMC2505A</td><td>fig|1502.2785.peg.3726</td><td></td><td></td><td>TTDI02058</td><td></td><td></td><td>Translation elongation factor Tu</td><td></td><td></td><td></td><td>5</td><td>88</td><td>86</td><td>3e-02</td></tr>
<tr><td>K-mer Search</td><td>Antibiotic Resistance</td><td>PATRIC</td><td>Clostridium perfringens IRMC2505A</td><td>fig|1502.2785.peg.1048</td><td></td><td></td><td></td><td></td><td>Iso-tRNA</td><td>Isoleucyl-tRNA synthetase (EC 6.1.1.5)</td><td>Isoleucyl-tRNA synthetase (EC 6.1.1.5)</td><td>antibiotic target in susceptible species</td><td>7929087</td><td></td><td></td><td></td><td></td></tr>
<tr><td>K-mer Search</td><td>Antibiotic Resistance</td><td>PATRIC</td><td>Clostridium perfringens IRMC2505A</td><td>fig|1502.2785.peg.1111</td><td></td><td></td><td></td><td></td><td>Alr</td><td>Alanine racemase (EC 5.1.1.1)</td><td>Alanine racemase (EC 5.1.1.1)</td><td>antibiotic target in susceptible species</td><td>19748470;24303782</td><td></td><td></td><td></td><td></td></tr>
<tr><td>BLAT</td><td>Antibiotic Resistance</td><td>CARD</td><td>Clostridium perfringens IRMC2505A</td><td>fig|1502.2785.peg.824</td><td></td><td></td><td>WP_009887863.1</td><td>Clostridium difficile</td><td></td><td>Translation elongation factor Tu</td><td></td><td>antibiotic resistant gene variant or mutant,elfamycin resistance gene</td><td></td><td>6</td><td>96</td><td>83</td><td>7e-03</td></tr>
<tr><td>BLAT</td><td>Virulence Factor</td><td>Victors</td><td>Clostridium perfringens IRMC2505A</td><td>fig|1502.2785.peg.710</td><td></td><td></td><td>110801372</td><td>Clostridium perfringens ATCC 13124</td><td>virS</td><td>hypothetical protein</td><td></td><td></td><td>8052128</td><td>99</td><td>100</td><td>95</td><td>1e-243</td></tr>
<tr><td>K-mer Search</td><td>Antibiotic Resistance</td><td>PATRIC</td><td>Clostridium perfringens IRMC2505A</td><td>fig|1502.2785.peg.824</td><td></td><td></td><td></td><td></td><td>EF-Tu</td><td>Translation elongation factor Tu</td><td>Translation elongation factor Tu</td><td>antibiotic target in susceptible species</td><td>364475;9678602</td><td></td><td></td><td></td><td></td></tr>
<tr><td>BLAT</td><td>Transporter</td><td>TCDB</td><td>Clostridium perfringens IRMC2505A</td><td>fig|1502.2785.peg.3983</td><td></td><td></td><td>Q46305</td><td>Clostridium perfringens</td><td></td><td>Tetracycline resistance, MFS efflux pump => TetA(P)</td><td>the major facilitator superfamily (mfs).</td><td>2.A.1.21.6</td><td>16788202;8170402</td><td>100</td><td>100</td><td>98</td><td>1e-234</td></tr>
<tr><td>BLAT</td><td>Antibiotic Resistance</td><td>NDARO</td><td>Clostridium perfringens IRMC2505A</td><td>fig|1502.2785.peg.3983</td><td></td><td></td><td>WP_003479690.1</td><td>Clostridium perfringens</td><td></td><td>Tetracycline resistance, MFS efflux pump => TetA(P)</td><td>tetracycline efflux MFS transporter TetA(P)</td><td></td><td></td><td>100</td><td>100</td><td>100</td><td>1e-237</td></tr>
<tr><td>BLAT</td><td>Virulence Factor</td><td>Victors</td><td>Clostridium perfringens IRMC2505A</td><td>fig|1502.2785.peg.2183</td><td></td><td></td><td>18309145</td><td>Clostridium perfringens str. 13</td><td>pfoA</td><td>Thiol-activated cytolysin</td><td></td><td></td><td>9182756</td><td>92</td><td>88</td><td>87</td><td>1e-243</td></tr>
<tr><td>K-mer Search</td><td>Antibiotic Resistance</td><td>PATRIC</td><td>Clostridium perfringens IRMC2505A</td><td>fig|1502.2785.peg.1362</td><td></td><td></td><td></td><td></td><td>FabK-like</td><td>Putative FabK-like enoyl-[acyl-carrier-protein] reductase</td><td>Putative FabK-like enoyl-[acyl-carrier-protein] reductase</td><td>protein involved in antibiotic sequestration</td><td>27577999</td><td></td><td></td><td></td><td></td></tr>
<tr><td>BLAT</td><td>Virulence Factor</td><td>Victors</td><td>Clostridium perfringens IRMC2505A</td><td>fig|1502.2785.peg.3726</td><td></td><td></td><td>15676067</td><td>Neisseria meningitidis MC58</td><td>tufA</td><td>Translation elongation factor Tu</td><td></td><td></td><td>11062540</td><td>5</td><td>92</td><td>86</td><td>3e-03</td></tr>
</table>

BLAT	Antibiotic Resistance	CARD	Clostridium perfringens IRMC2505A	fig|1502.2785.peg.812			Q0SSM7	Clostridium perfringens SM101	mprF	putative membrane protein		antibiotic target modifying enzyme,peptide antibiotic resistance gene		100	100	95	0.0
K-mer Search	Antibiotic Resistance	PATRIC	Clostridium perfringens IRMC2505A	fig|1502.2785.peg.4118					NimB	Nitroimidazole resistance protein NimB	Nitroimidazole resistance protein NimB	antibiotic inactivation enzyme	16338949				
K-mer Search	Antibiotic Resistance	PATRIC	Clostridium perfringens IRMC2505A	fig|1502.2785.peg.2790					gyrB	DNA gyrase subunit B (EC 5.99.1.3)	DNA gyrase subunit B (EC 5.99.1.3)	antibiotic target in susceptible species	21693461;22279180;9293187				
BLAT	Transporter	TCDB	Clostridium perfringens IRMC2505A	fig|1502.2785.peg.3028			Q8XME6	Clostridium perfringens (strain 13 / Type A)		Carbon starvation protein A	the putative peptide transporter carbon starvation csta (csta) family.	2.A.114.1.4		100	100	99	1e-277
BLAT	Virulence Factor	VFDB	Clostridium perfringens IRMC2505A	fig|1502.2785.peg.1125			VFG002278	Clostridium perfringens str. 13	nagI	Protein O-GlcNAcase (EC 3.2.1.169)		Exoenzyme,Carbohydrate-active enzyme,Hyaluronidase		100	100	96	0.0
BLAT	Drug Target	DrugBank	Clostridium perfringens IRMC2505A	fig|1502.2785.peg.3726			P60339	Thermus thermophilus (strain HB8 / ATCC 27634 / DSM 579)	tufB	Translation elongation factor Tu	Aurodox;Guanosine-5'-Diphosphate	Drug target		5	96	87	9e-04
K-mer Search	Antibiotic Resistance	PATRIC	Clostridium perfringens IRMC2505A	fig|1502.2785.peg.3986					TetB(P)	Tetracycline resistance, ribosomal protection type => TetB(P)	Tetracycline resistance, ribosomal protection type => TetB(P)	antibiotic target protection protein	8170402;11717269				
K-mer Search	Antibiotic Resistance	PATRIC	Clostridium perfringens IRMC2505A	fig|1502.2785.peg.825					S10p	SSU ribosomal protein S10p (S20e)	SSU ribosomal protein S10p (S20e)	antibiotic target in susceptible species	26124155				
BLAT	Transporter	TCDB	Clostridium perfringens IRMC2505A	fig|1502.2785.peg.2308			B1V6H1	Clostridium perfringens D str. JGS1721		hypothetical protein	the 11 holin (11 holin) family.	1.E.11.2.2		100	100	87	2e-35
K-mer Search	Antibiotic Resistance	PATRIC	Clostridium perfringens IRMC2505A	fig|1502.2785.peg.314					GdpD	Glycerophosphoryl diester phosphodiesterase (EC 3.1.4.46)	Glycerophosphoryl diester phosphodiesterase (EC 3.1.4.46)	protein altering cell wall charge conferring antibiotic resistance	21899450				
K-mer Search	Antibiotic Resistance	PATRIC	Clostridium perfringens IRMC2505A	fig|1502.2785.peg.1498					kasA	3-oxoacyl-[acyl-carrier-protein] synthase, KASII (EC 2.3.1.179)	3-oxoacyl-[acyl-carrier-protein] synthase, KASII (EC 2.3.1.179)	antibiotic target in susceptible species	10428945				
BLAT	Virulence Factor	Victors	Clostridium perfringens IRMC2505A	fig|1502.2785.peg.1147			29376182	Enterococcus faecalis V583	EF1623	Ethanolamine utilization protein similar to PduA/PduJ			17307944	91	91	92	4e-38
BLAT	Virulence Factor	VFDB	Clostridium perfringens IRMC2505A	fig|1502.2785.peg.3754			VFG002274	Clostridium perfringens str. 13	plc	Broad-substrate range phospholipase C (EC 3.1.4.3)		Toxin,Zinc-metallophospholipase C		100	100	99	1e-242
BLAT	Antibiotic Resistance	CARD	Clostridium perfringens IRMC2505A	fig|1502.2785.peg.3983			AAA20116.1	Clostridium perfringens	tetA(P)	Tetracycline resistance, MFS efflux pump => TetA(P)		efflux pump conferring antibiotic resistance		100	100	98	1e-234
K-mer Search	Antibiotic Resistance	PATRIC	Clostridium perfringens IRMC2505A	fig|1502.2785.peg.513					fabV	Enoyl-[acyl-carrier-protein] reductase [NADH] (EC 1.3.1.9), FabV => refractory to triclosan	Enoyl-[acyl-carrier-protein] reductase [NADH] (EC 1.3.1.9), FabV => refractory to triclosan	antibiotic target replacement protein	19933806;18032386				
BLAT	Virulence Factor	VFDB	Clostridium perfringens IRMC2505A	fig|1502.2785.peg.2129			VFG002282	Clostridium perfringens str. 13	cloSI	hypothetical protein		Exoenzyme,Cysteine endopeptidase		99	100	97	1e-306
K-mer Search	Antibiotic Resistance	PATRIC	Clostridium perfringens IRMC2505A	fig|1502.2785.peg.3718					MurA	UDP-N-acetylglucosamine 1-carboxyvinyltransferase (EC 2.5.1.7)	UDP-N-acetylglucosamine 1-carboxyvinyltransferase (EC 2.5.1.7)	antibiotic target in susceptible species	8994972				
BLAT	Antibiotic Resistance	CARD	Clostridium perfringens IRMC2505A	fig|1502.2785.peg.3726			WP_009887863.1	Clostridium difficile		Translation elongation factor Tu		antibiotic resistant gene variant or mutant,elfamycin resistance gene		6	96	83	7e-03
BLAT	Antibiotic Resistance	CARD	Clostridium perfringens IRMC2505A	fig|1502.2785.peg.3987			AAA20117.1	Clostridium perfringens	tetB(P)	Tetracycline resistance, ribosomal protection type => TetB(P)		antibiotic target protection protein,tetracycline resistance gene		48	99	98	1e-180
K-mer Search	Antibiotic Resistance	PATRIC	Clostridium perfringens IRMC2505A	fig|1502.2785.peg.3983					TetA(P)	Tetracycline resistance, MFS efflux pump => TetA(P)	Tetracycline resistance, MFS efflux pump => TetA(P)	efflux pump conferring antibiotic resistance	8170402;11717269				
BLAT	Virulence Factor	VFDB	Clostridium perfringens IRMC2505A	fig|1502.2785.peg.4032			VFG002279	Clostridium perfringens str. 13	nagJ	Protein O-GlcNAcase (EC 3.2.1.169)		Exoenzyme,Carbohydrate-active enzyme,Hyaluronidase		100	100	98	0.0
K-mer Search	Antibiotic Resistance	PATRIC	Clostridium perfringens IRMC2505A	fig|1502.2785.peg.80					dxr	1-deoxy-D-xylulose 5-phosphate reductoisomerase (EC 1.1.1.267)	1-deoxy-D-xylulose 5-phosphate reductoisomerase (EC 1.1.1.267)	antibiotic target in susceptible species	16321944				
K-mer Search	Antibiotic Resistance	PATRIC	Clostridium perfringens IRMC2505A	fig|1502.2785.peg.1695					MprF	L-O-lysylphosphatidylglycerol synthase (EC 2.3.2.3)	L-O-lysylphosphatidylglycerol synthase (EC 2.3.2.3)	protein altering cell wall charge conferring antibiotic resistance	19289517;16723576				
BLAT	Drug Target	DrugBank	Clostridium perfringens IRMC2505A	fig|1502.2785.peg.399			Q8XJ01	Clostridium perfringens (strain 13 / Type A)	pbpA	Multimodular transpeptidase-transglycosylase (EC 2.4.1.129) (EC 3.4.-.-)	Cefotiam;Cefmenoxime;Flucloxacillin;Phenoxymethylpenicillin;Loracarbef;Cefalotin;Oxacillin;Cefaclor;Mezlocillin;Cyclacillin;Amoxicillin;Azlocillin;Cefditoren;Cefuroxime;Cefapirin;Cloxacillin;Bacampicillin;Pivampicillin;Pivmecillinam;Degraded Cephaloridine	Drug target		99	81	97	0.0
BLAT	Drug Target	DrugBank	Clostridium perfringens IRMC2505A	fig|1502.2785.peg.824			P60339	Thermus thermophilus (strain HB8 / ATCC 27634 / DSM 579)	tufB	Translation elongation factor Tu	Aurodox;Guanosine-5'-Diphosphate	Drug target		5	96	87	9e-04
BLAT	Antibiotic Resistance	NDARO	Clostridium perfringens IRMC2505A	fig|1502.2785.peg.3987			WP_012478245.1	Clostridium perfringens		Tetracycline resistance, ribosomal protection type => TetB(P)	tetracycline resistance ribosomal protection protein TetB(P)			48	99	98	1e-180

K-mer Search	Antibiotic Resistance	PATRIC	Clostridium perfringens IRMC2505A	fig|1502.2785.peg.646					rho	Transcription termination factor Rho	Transcription termination factor Rho	antibiotic target in susceptible species	8466900				
K-mer Search	Antibiotic Resistance	PATRIC	Clostridium perfringens IRMC2505A	fig|1502.2785.peg.2791					gyrA	DNA gyrase subunit A (EC 5.99.1.3)	DNA gyrase subunit A (EC 5.99.1.3)	antibiotic target in susceptible species	9293187				
K-mer Search	Antibiotic Resistance	PATRIC	Clostridium perfringens IRMC2505A	fig|1502.2785.peg.1881					GdpD	Glycerophosphoryl diester phosphodiesterase (EC 3.1.4.46)	Glycerophosphoryl diester phosphodiesterase (EC 3.1.4.46)	protein altering cell wall charge conferring antibiotic resistance	21899450				
K-mer Search	Antibiotic Resistance	PATRIC	Clostridium perfringens IRMC2505A	fig|1502.2785.peg.60					PgsA	CDP-diacylglycerol--glycerol-3-phosphate 3-phosphatidyltransferase (EC 2.7.8.5)	CDP-diacylglycerol--glycerol-3-phosphate 3-phosphatidyltransferase (EC 2.7.8.5)	protein altering cell wall charge conferring antibiotic resistance	22238576				
K-mer Search	Antibiotic Resistance	PATRIC	Clostridium perfringens IRMC2505A	fig|1502.2785.peg.2776					gidB	16S rRNA (guanine(527)-N(7))-methyltransferase (EC 2.1.1.170)	16S rRNA (guanine(527)-N(7))-methyltransferase (EC 2.1.1.170)	gene conferring resistance via absence	17238915				
BLAT	Virulence Factor	Victors	Clostridium perfringens IRMC2505A	fig|1502.2785.peg.3754			18309018	Clostridium perfringens str. 13	plc	Broad-substrate range phospholipase C (EC 3.1.4.3)			7746141	100	100	99	1e-242
BLAT	Transporter	TCDB	Clostridium perfringens IRMC2505A	fig|1502.2785.peg.3795			Q0SPM4	Clostridium perfringens (strain SM101 / Type A)		bacteriocin UviB	the bhla holin (bhla holin) family.	1.E.27.1.5		95	92	98	3e-26
K-mer Search	Antibiotic Resistance	PATRIC	Clostridium perfringens IRMC2505A	fig|1502.2785.peg.3343					gyrA	DNA gyrase subunit A (EC 5.99.1.3)	DNA gyrase subunit A (EC 5.99.1.3)	antibiotic target in susceptible species	9293187				
BLAT	Virulence Factor	VFDB	Clostridium perfringens IRMC2505A	fig|1502.2785.peg.736			VFG002281	Clostridium perfringens str. 13	nagL	hypothetical protein		Exoenzyme,Carbohydrate-active enzyme,Hyaluronidase		100	100	95	0.0
K-mer Search	Antibiotic Resistance	PATRIC	Clostridium perfringens IRMC2505A	fig|1502.2785.peg.3735					rpoC	DNA-directed RNA polymerase beta' subunit (EC 2.7.7.6)	DNA-directed RNA polymerase beta' subunit (EC 2.7.7.6)	antibiotic target in susceptible species	16723576				
BLAT	Virulence Factor	VFDB	Clostridium perfringens IRMC2505A	fig|1502.2785.peg.3010			VFG002283	Clostridium perfringens str. 13	nanI	Sialidase (EC 3.2.1.18)		Exoenzyme,Carbohydrate-active enzyme,Sialidase		100	100	99	0.0
BLAT	Virulence Factor	VFDB	Clostridium perfringens IRMC2505A	fig|1502.2785.peg.2195			VFG002276	Clostridium perfringens str. 13	colA	Microbial collagenase (EC 3.4.24.3)		Exoenzyme,Collagenase		100	100	98	0.0
K-mer Search	Antibiotic Resistance	PATRIC	Clostridium perfringens IRMC2505A	fig|1502.2785.peg.3984					TetB(P)	Tetracycline resistance, ribosomal protection type => TetB(P)	Tetracycline resistance, ribosomal protection type => TetB(P)	antibiotic target protection protein	8170402;11717269				
K-mer Search	Antibiotic Resistance	PATRIC	Clostridium perfringens IRMC2505A	fig|1502.2785.peg.2868					MurA	UDP-N-acetylglucosamine 1-carboxyvinyltransferase (EC 2.5.1.7)	UDP-N-acetylglucosamine 1-carboxyvinyltransferase (EC 2.5.1.7)	antibiotic target in susceptible species	8994972				
BLAT	Antibiotic Resistance	CARD	Clostridium perfringens IRMC2505A	fig|1502.2785.peg.3985			AAA20117.1	Clostridium perfringens	tetB(P)	Tetracycline resistance, ribosomal protection type => TetB(P)		antibiotic target protection protein,tetracycline resistance gene		13	91	95	5e-40
BLAT	Drug Target	DrugBank	Clostridium perfringens IRMC2505A	fig|1502.2785.peg.2494			P54965	Clostridium perfringens (strain 13 / Type A)	cbh	Choloylglycine hydrolase (EC 3.5.1.24)	2-Aminoethanesulfonic Acid;Cholic Acid;Deoxycholic Acid	Drug target		100	100	91	1e-182
K-mer Search	Antibiotic Resistance	PATRIC	Clostridium perfringens IRMC2505A	fig|1502.2785.peg.2322					folP	Dihydropteroate synthase (EC 2.5.1.15)	Dihydropteroate synthase (EC 2.5.1.15)	antibiotic target in susceptible species	15673783				
K-mer Search	Antibiotic Resistance	PATRIC	Clostridium perfringens IRMC2505A	fig|1502.2785.peg.2156					Ddl	D-alanine--D-alanine ligase (EC 6.3.2.4)	D-alanine--D-alanine ligase (EC 6.3.2.4)	antibiotic target in susceptible species	24303782;24033232				
BLAT	Virulence Factor	VFDB	Clostridium perfringens IRMC2505A	fig|1502.2785.peg.2700			VFG002284	Clostridium perfringens str. 13	nanJ	Sialidase (EC 3.2.1.18)		Exoenzyme,Carbohydrate-active enzyme,Sialidase		100	100	95	0.0
BLAT	Transporter	TCDB	Clostridium perfringens IRMC2505A	fig|1502.2785.peg.3198			Q186B7	Clostridium difficile (strain 630)		Sulfite transporter, NirC family	the formate-nitrite transporter (fnt) family.	1.A.16.3.3		62	99	100	1e-85
BLAT	Virulence Factor	VFDB	Clostridium perfringens IRMC2505A	fig|1502.2785.peg.2183			VFG002275	Clostridium perfringens str. 13	pfoA	Thiol-activated cytolysin		Toxin,Membrane-damaging,Pore-forming,Channel-forming involving beta-sheet-containing toxin (beta-barrel),Cholesterol-dependent cytolysin		92	88	87	1e-243
BLAT	Virulence Factor	VFDB	Clostridium perfringens IRMC2505A	fig|1502.2785.peg.2213			VFG002277	Clostridium perfringens str. 13	nagH	Hyaluronoglucosaminidase (EC 3.2.1.35)		Exoenzyme,Carbohydrate-active enzyme,Hyaluronidase		100	100	97	0.0
K-mer Search	Antibiotic Resistance	PATRIC	Clostridium perfringens IRMC2505A	fig|1502.2785.peg.1762					Ddl	D-alanine--D-alanine ligase (EC 6.3.2.4)	D-alanine--D-alanine ligase (EC 6.3.2.4)	antibiotic target in susceptible species	24303782;24033232				
BLAT	Transporter	TCDB	Clostridium perfringens IRMC2505A	fig|1502.2785.peg.2585			P50487	Clostridium perfringens		uracil-xanthine permease	the nucleobase/ascorbate transporter (nat) or nucleobase:cation symporter-2 (ncs2) family.	2.A.40.2.1	11792842;8162194	100	100	99	1e-250
K-mer Search	Antibiotic Resistance	PATRIC	Clostridium perfringens IRMC2505A	fig|1502.2785.peg.2657					Alr	Alanine racemase (EC 5.1.1.1)	Alanine racemase (EC 5.1.1.1)	antibiotic target in susceptible species	19748470;24303782				
K-mer Search	Antibiotic Resistance	PATRIC	Clostridium perfringens IRMC2505A	fig|1502.2785.peg.3429					folA, Dfr	Dihydrofolate reductase (EC 1.5.1.3)	Dihydrofolate reductase (EC 1.5.1.3)	antibiotic target in susceptible species	20169085;25288078				
BLAT	Transporter	TCDB	Clostridium perfringens IRMC2505A	fig|1502.2785.peg.1109			H7CZV2	Clostridium perfringens F262		hypothetical protein	the mycobacterial 4 tms phage holin (mp4 holin) family.	1.E.40.4.3		100	100	97	1e-60
K-mer Search	Antibiotic Resistance	PATRIC	Clostridium perfringens IRMC2505A	fig|1502.2785.peg.3734					rpoB	DNA-directed RNA polymerase beta subunit (EC 2.7.7.6)	DNA-directed RNA polymerase beta subunit (EC 2.7.7.6)	antibiotic target in susceptible species	3050121;15047531;16723576				

Supplementary Table S4: Gene families are ranked by alignment score for phylogenetic tree of IRMC2505A genome.

PGFam	Align. Score	Align. Length	Num Seqs	Mean Sqr Freq	Prop Gaps	Used In Analysis	Product
PGF_02797402	27.70	1333	22	0.759	0.070	True	DNA-directed RNA polymerase beta subunit (EC 2.7.7.6)
PGF_02704551	27.20	1269	22	0.764	0.071	True	DNA-directed RNA polymerase beta' subunit (EC 2.7.7.6)
PGF_05171623	22.81	1088	22	0.692	0.045	True	Isoleucyl-tRNA synthetase (EC 6.1.1.5)
PGF_05500127	21.83	903	22	0.726	0.024	True	Valyl-tRNA synthetase (EC 6.1.1.9)
PGF_00950554	19.48	674	22	0.750	0.022	True	Excinuclease ABC subunit B
PGF_02906681	19.10	732	22	0.706	0.016	True	Transcription accessory protein (S1 RNA-binding domain)
PGF_03104485	18.87	752	22	0.688	0.062	True	Polyribonucleotide nucleotidyltransferase (EC 2.7.7.8)
PGF_00033095	18.36	817	22	0.642	0.029	True	Phenylalanyl-tRNA synthetase beta chain (EC 6.1.1.20)
PGF_10300474	18.13	714	22	0.679	0.025	True	DNA topoisomerase I (EC 5.99.1.2)
PGF_00030643	17.91	541	22	0.770	0.019	True	Peptide chain release factor 3
PGF_06776852	17.37	804	22	0.612	0.022	True	Recombination inhibitory protein MutS2
PGF_00057270	17.26	1020	22	0.541	0.076	True	DNA topoisomerase IV subunit A (EC 5.99.1.3)
PGF_09950730	17.26	1197	22	0.499	0.009	True	Chromosome partition protein smc
PGF_03752158	17.00	635	22	0.674	0.027	True	FIG092679: Fe-S oxidoreductase
PGF_00047095	16.34	811	22	0.574	0.081	True	RecD-like DNA helicase YrrC
PGF_00021217	16.31	518	22	0.716	0.013	True	2,3-bisphosphoglycerate-independent phosphoglycerate mutase (EC 5.4.2.12)
PGF_10525419	16.23	923	22	0.534	0.243	True	Translation initiation factor 2
PGF_00075770	16.17	454	22	0.759	0.006	True	FIG00519347: Ribonucleotide reductase-like protein
PGF_00066502	15.95	467	22	0.738	0.010	True	Asparaginyl-tRNA synthetase (EC 6.1.1.22)
PGF_02277678	15.78	406	22	0.783	0.023	True	Phosphoglycerate kinase (EC 2.7.2.3)
PGF_00025600	15.78	500	22	0.706	0.017	True	Nicotinate phosphoribosyltransferase (EC 6.3.4.21)
PGF_00007024	15.60	442	22	0.742	0.008	True	GTP-binding protein EngA
PGF_00052238	15.24	474	22	0.700	0.049	True	Signal recognition particle protein Ffh
PGF_01137124	14.94	465	22	0.693	0.033	True	Phosphoglucosamine mutase (EC 5.4.2.10)

PGF_00007012	14.89	371	22	0.773	0.016	True	GTP-binding and nucleic acid-binding protein YchF
PGF_02226715	14.86	705	22	0.560	0.036	True	ATP-dependent DNA helicase RecG (EC 3.6.4.12)
PGF_00030640	14.55	363	22	0.763	0.012	True	Peptide chain release factor 1
PGF_00011081	14.47	853	22	0.495	0.134	True	Helicase PriA essential for oriC/DnaA-independent DNA replication
PGF_01281052	14.45	856	22	0.494	0.086	True	Uncharacterized protease CPF_2136
PGF_02029783	14.40	443	22	0.684	0.036	True	GTP-binding protein Obg
PGF_00422271	14.39	320	22	0.805	0.015	True	DNA-directed RNA polymerase alpha subunit (EC 2.7.7.6)
PGF_00060120	14.24	467	22	0.659	0.027	True	SCIFF radical SAM maturase
PGF_00024314	14.20	571	22	0.594	0.064	True	NAD(FAD)-utilizing dehydrogenase, sll0175 homolog
PGF_02019462	13.89	341	22	0.752	0.006	True	Phenylalanyl-tRNA synthetase alpha chain (EC 6.1.1.20)
PGF_00019248	13.68	553	22	0.582	0.012	True	Manganese-dependent inorganic pyrophosphatase (EC 3.6.1.1)
PGF_08562657	13.67	512	22	0.604	0.118	True	tRNA-i(6)A37 methylthiotransferase (EC 2.8.4.3)
PGF_00063999	13.67	464	22	0.634	0.012	True	UDP-N-acetylmuramate--L-alanine ligase (EC 6.3.2.8)
PGF_05049118	13.56	358	22	0.717	0.039	True	Holliday junction ATP-dependent DNA helicase RuvB (EC 3.6.4.12)
PGF_03520151	13.54	419	22	0.661	0.102	True	Cell division protein FtsZ
PGF_00016393	13.49	281	22	0.804	0.015	True	LSU ribosomal protein L2p (L8e)
PGF_00007028	13.30	626	22	0.531	0.086	True	Ribosome LSU-associated GTP-binding protein HflX
PGF_09111052	13.25	407	22	0.657	0.028	True	Phosphopantothenoylcysteine decarboxylase (EC 4.1.1.36) / Phosphopantothenoylcysteine synthetase (EC 6.3.2.5)
PGF_09945387	13.13	600	22	0.536	0.038	True	Fibronectin/fibrinogen-binding protein
PGF_00426236	13.08	359	22	0.690	0.027	True	(E)-4-hydroxy-3-methylbut-2-enyl-diphosphate synthase (flavodoxin) (EC 1.17.7.3)
PGF_02620298	13.03	472	22	0.600	0.042	True	Argininosuccinate lyase (EC 4.3.2.1)
PGF_00048846	12.91	470	22	0.596	0.046	True	Ribosomal protein S12p Asp88 (E. coli) methylthiotransferase (EC 2.8.4.4)
PGF_09019918	12.77	438	22	0.610	0.025	True	Glutamate-1-semialdehyde 2,1-aminomutase (EC 5.4.3.8)
PGF_00421796	12.71	579	22	0.528	0.025	True	DNA repair protein RecN
PGF_00413298	12.64	487	22	0.573	0.087	True	tRNA t(6)A37-methylthiotransferase (EC 2.8.4.5)
PGF_00033444	12.61	716	22	0.471	0.087	True	Cyclic-di-AMP phosphodiesterase GdpP
PGF_07063065	12.50	436	22	0.598	0.158	True	Transcription termination protein NusA
PGF_04505269	12.38	246	22	0.790	0.050	True	SSU ribosomal protein S2p (SAe)

PGF_05507938	12.36	794	22	0.439	0.184	True	DNA mismatch repair protein MutL
PGF_00004169	12.26	711	22	0.460	0.205	True	Fe-S oxidoreductase
PGF_02923127	12.17	240	22	0.785	0.014	True	Uridylate kinase (EC 2.7.4.22)
PGF_03788368	12.02	296	22	0.699	0.008	True	RNase adapter protein RapZ
PGF_00618776	11.89	361	22	0.626	0.038	True	23S rRNA (adenine(2503)-C(2))-methyltransferase @ tRNA (adenine(37)-C(2))-methyltransferase (EC 2.1.1.192)
PGF_00007027	11.84	306	22	0.677	0.034	True	GTP-binding protein Era
PGF_03295331	11.80	374	22	0.610	0.044	True	UDP-N-acetylglucosamine--N-acetylmuramyl-(pentapeptide) pyrophosphoryl-undecaprenol N-acetylglucosamine transferase (EC 2.4.1.227)
PGF_00415407	11.77	392	22	0.595	0.032	True	23S rRNA (guanine(2445)-N(2))-methyltransferase (EC 2.1.1.173)
PGF_00049889	11.74	230	22	0.774	0.035	True	SSU ribosomal protein S3p (S3e)
PGF_00057483	11.63	819	22	0.406	0.385	True	Transcription termination factor Rho
PGF_06936561	11.47	313	22	0.648	0.038	True	Translation elongation factor Ts
PGF_00769755	11.37	456	22	0.532	0.033	True	16S rRNA (cytosine(967)-C(5))-methyltransferase (EC 2.1.1.176)
PGF_00403095	11.34	211	22	0.781	0.009	True	Uracil phosphoribosyltransferase (EC 2.4.2.9)
PGF_00426877	11.24	299	22	0.650	0.052	True	LSU ribosomal maturation GTPase RbgA (B. subtilis YlqF)
PGF_00016443	11.14	181	22	0.828	0.008	True	LSU ribosomal protein L5p (L11e)
PGF_05767868	11.09	250	22	0.701	0.020	True	Probable transcriptional regulatory protein YebC
PGF_00011463	11.05	286	22	0.654	0.053	True	RNA binding methyltransferase FtsJ like
PGF_00418291	11.02	266	22	0.676	0.049	True	Cobalt-precorrin-4 C(11)-methyltransferase (EC 2.1.1.271)
PGF_00007045	10.72	304	22	0.615	0.145	True	GTP-sensing transcriptional pleiotropic repressor CodY
PGF_04807486	10.62	335	22	0.580	0.070	True	tRNA dimethylallyltransferase (EC 2.5.1.75)
PGF_00049896	10.59	169	22	0.814	0.022	True	SSU ribosomal protein S5p (S2e)
PGF_00049904	10.59	156	22	0.848	0.000	True	SSU ribosomal protein S7p (S5e)
PGF_04512522	10.58	208	22	0.734	0.017	True	LSU ribosomal protein L4p (L1e)
PGF_06230969	10.48	553	22	0.446	0.092	True	NAD(P)H-hydrate epimerase (EC 5.1.99.6) / ADP-dependent (S)-NAD(P)H-hydrate dehydratase (EC 4.2.1.136)
PGF_00425024	10.41	433	22	0.500	0.075	True	Exodeoxyribonuclease VII large subunit (EC 3.1.11.6)
PGF_00286985	10.39	462	22	0.483	0.111	True	[FeFe]-hydrogenase maturation protein HydF
PGF_09155108	10.32	444	22	0.490	0.092	True	Exonuclease SbcD

PGF_00413208	10.25	269	22	0.625	0.107	True	tRNA (guanine(37)-N(1))-methyltransferase (EC 2.1.1.228)
PGF_06461498	10.20	318	22	0.572	0.087	True	Orotidine 5'-phosphate decarboxylase (EC 4.1.1.23)
PGF_00046555	10.14	234	22	0.663	0.089	True	RNA polymerase sporulation specific sigma factor SigH
PGF_07695531	10.08	141	22	0.849	0.000	True	LSU ribosomal protein L11p (L12e)
PGF_00047155	10.07	217	22	0.684	0.029	True	Redox-sensing transcriptional repressor Rex
PGF_04244475	10.07	234	22	0.659	0.074	True	Adenylate kinase (EC 2.7.4.3)
PGF_00016444	10.02	181	22	0.745	0.008	True	LSU ribosomal protein L6p (L9e)
PGF_01212500	9.90	307	22	0.565	0.088	True	Diadenylate cyclase spyDAC; Bacterial checkpoint controller DisA with nucleotide-binding domain
PGF_00423533	9.90	316	22	0.557	0.104	True	4-diphosphocytidyl-2-C-methyl-D-erythritol kinase (EC 2.7.1.148)
PGF_02746079	9.88	326	22	0.547	0.092	True	Transcription regulator [contains diacylglycerol kinase catalytic domain]
PGF_01867628	9.86	392	22	0.498	0.116	True	Heat-inducible transcription repressor HrcA
PGF_00000196	9.77	446	22	0.462	0.091	True	UPF0348 protein family
PGF_02472178	9.71	277	22	0.583	0.068	True	Uncharacterized metal-dependent hydrolase YcfH
PGF_00049909	9.66	131	22	0.844	0.007	True	SSU ribosomal protein S9p (S16e)
PGF_06784545	9.64	304	22	0.553	0.053	True	(2E,6E)-farnesyl diphosphate synthase (EC 2.5.1.10)
PGF_00689961	9.63	237	22	0.626	0.110	True	Guanylate kinase (EC 2.7.4.8)
PGF_06941403	9.59	139	22	0.814	0.091	True	SSU ribosomal protein S12p (S23e)
PGF_00418285	9.56	287	22	0.565	0.151	True	Cobalt-precorrin-3 C(17)-methyltransferase (EC 2.1.1.272)
PGF_10396914	9.55	319	22	0.535	0.072	True	Similar to ribosomal large subunit pseudouridine synthase D, Bacillus subtilis YjbO type
PGF_08582746	9.49	318	22	0.532	0.128	True	23S rRNA (guanosine(2251)-2'-O)-methyltransferase (EC 2.1.1.185)
PGF_06377994	9.45	544	22	0.405	0.265	True	FtsW-like cell division membrane protein CA_C0505
PGF_07102049	9.42	309	22	0.536	0.050	False	UPF0701 protein YicC
PGF_02623406	9.31	318	22	0.522	0.026	False	FMN adenylyltransferase (EC 2.7.7.2) / Riboflavin kinase (EC 2.7.1.26)
PGF_03174068	9.28	186	22	0.680	0.062	False	Transcription antitermination protein NusG
PGF_00035334	9.22	342	22	0.498	0.066	False	Adenosylcobinamide-phosphate synthase (EC 6.3.1.10)
PGF_03790040	9.07	256	22	0.567	0.083	False	Ribonuclease III (EC 3.1.26.3)
PGF_00049906	9.07	133	22	0.786	0.008	False	SSU ribosomal protein S8p (S15Ae)

PGF_00413295	8.98	339	22	0.488	0.135	False	tRNA pseudouridine(55) synthase (EC 5.4.99.25)
PGF_00418309	8.89	238	22	0.576	0.121	False	Cobalt-precorrin-8 methylmutase (EC 5.4.99.60)
PGF_00049840	8.88	126	22	0.791	0.028	False	SSU ribosomal protein S13p (S18e)
PGF_00413554	8.83	159	22	0.700	0.019	False	tmRNA-binding protein SmpB
PGF_03295678	8.81	199	22	0.624	0.041	False	CDP-diacylglycerol--glycerol-3-phosphate 3-phosphatidyltransferase (EC 2.7.8.5)
PGF_01833449	8.68	343	22	0.468	0.074	False	3'-to-5' oligoribonuclease A, Bacillus type
PGF_00016445	8.58	126	22	0.764	0.036	False	LSU ribosomal protein L7p/L12p (P1/P2)
PGF_06530721	8.57	228	22	0.567	0.058	False	tRNA (guanine(46)-N(7))-methyltransferase (EC 2.1.1.33)
PGF_02455692	8.56	237	22	0.556	0.072	False	Cytidylate kinase (EC 2.7.4.25)
PGF_04486278	8.34	340	22	0.452	0.057	False	Biotin operon repressor / Biotin--protein ligase (EC 6.3.4.9)(EC 6.3.4.10)(EC 6.3.4.11)(EC 6.3.4.15)
PGF_00717752	8.31	250	22	0.526	0.105	False	Cobalt-precorrin-2 C(20)-methyltransferase (EC 2.1.1.151)
PGF_01213071	8.30	187	22	0.607	0.104	False	LSU ribosomal protein L10p (P0)
PGF_04788810	8.30	206	22	0.578	0.079	False	Peptidyl-tRNA hydrolase (EC 3.1.1.29)
PGF_00048556	8.28	299	22	0.479	0.107	False	Ribonuclease HII (EC 3.1.26.4)
PGF_00016385	8.24	101	22	0.820	0.018	False	LSU ribosomal protein L27p
PGF_04845029	8.18	120	22	0.747	0.039	False	LSU ribosomal protein L19p
PGF_07411046	8.16	290	22	0.479	0.116	False	Uncharacterized RNA methyltransferase YsgA
PGF_00016452	8.11	149	22	0.665	0.011	False	LSU ribosomal protein L9p
PGF_00007026	8.09	250	22	0.512	0.200	False	GTP-binding protein EngB
PGF_01382519	8.08	190	22	0.586	0.077	False	Ribosome hibernation promoting factor Hpf
PGF_03021159	8.05	94	22	0.830	0.045	False	UPF0296 protein YlzA
PGF_04762552	7.97	169	22	0.613	0.040	False	Phosphopantetheine adenylyltransferase (EC 2.7.7.3)
PGF_03189552	7.93	223	22	0.531	0.109	False	Holliday junction ATP-dependent DNA helicase RuvA (EC 3.6.4.12)
PGF_03753407	7.85	164	22	0.613	0.068	False	tRNA threonylcarbamoyladenosine biosynthesis protein TsaE
PGF_00049854	7.82	85	22	0.849	0.011	False	SSU ribosomal protein S17p (S11e)
PGF_04978890	7.82	104	22	0.767	0.008	False	LSU ribosomal protein L21p
PGF_00020361	7.81	170	22	0.599	0.026	False	Metal-dependent hydrolase YbeY, involved in rRNA and/or ribosome maturation and assembly
PGF_00413203	7.81	184	22	0.576	0.147	False	tRNA (cytidine(34)-2'-O)-methyltransferase (EC 2.1.1.207)

PGF_00060431	7.71	72	22	0.909	0.000	False	Translation initiation factor 1
PGF_07504595	7.58	153	22	0.613	0.066	False	3-hydroxyacyl-[acyl-carrier-protein] dehydratase, FabZ form (EC 4.2.1.59)
PGF_00413189	7.56	244	22	0.484	0.061	False	tRNA (adenine(22)-N(1))-methyltransferase (EC 2.1.1.217)
PGF_05579323	7.53	444	22	0.357	0.185	False	L-threonine 3-O-phosphate decarboxylase (EC 4.1.1.81)
PGF_00049860	7.42	102	22	0.735	0.083	False	SSU ribosomal protein S19p (S15e)
PGF_00422085	7.39	101	22	0.735	0.071	False	DNA-binding protein SpoVG
PGF_05636025	7.33	119	22	0.672	0.028	False	FIG001802: Putative alkaline-shock protein
PGF_00049847	7.33	88	22	0.781	0.011	False	SSU ribosomal protein S15p (S13e)
PGF_06649360	7.23	230	22	0.477	0.155	False	Segregation and condensation protein B
PGF_00016377	7.19	108	22	0.691	0.032	False	LSU ribosomal protein L24p (L26e)
PGF_00049901	7.13	97	22	0.724	0.019	False	SSU ribosomal protein S6p
PGF_00418278	7.13	498	22	0.319	0.310	False	Cobalt-precorrin 5A hydrolase (EC 3.7.1.12)
PGF_03990071	6.84	105	22	0.667	0.069	False	LSU ribosomal protein L23p (L23Ae)
PGF_00049842	6.79	61	22	0.870	0.000	False	SSU ribosomal protein S14p (S29e) @ SSU ribosomal protein S14p (S29e), zinc-dependent
PGF_08421732	6.75	343	22	0.365	0.179	False	NAD kinase (EC 2.7.1.23)
PGF_00178044	6.66	86	22	0.718	0.043	False	SSU ribosomal protein S16p
PGF_03818138	6.61	65	22	0.820	0.001	False	LSU ribosomal protein L35p
PGF_01135236	6.58	236	22	0.428	0.144	False	Dephospho-CoA kinase (EC 2.7.1.24)
PGF_02454577	6.58	88	22	0.701	0.009	False	SSU ribosomal protein S20p
PGF_00013347	6.46	97	22	0.656	0.128	False	UPF0297 protein YrzL
PGF_05770273	6.41	70	22	0.766	0.019	False	LSU ribosomal protein L31p @ LSU ribosomal protein L31p, zinc-dependent
PGF_06948903	6.40	95	22	0.656	0.116	False	SSU ribosomal protein S18p @ SSU ribosomal protein S18p, zinc-dependent
PGF_00415631	6.32	93	22	0.656	0.046	False	COG2740: Predicted nucleic-acid-binding protein implicated in transcription termination
PGF_02899131	6.27	72	22	0.739	0.037	False	LSU ribosomal protein L29p (L35e)
PGF_00598888	6.19	160	22	0.489	0.128	False	Mini-ribonuclease III
PGF_00011792	5.99	127	22	0.531	0.194	False	His repressor

PGF_06609275	5.83	49	22	0.833	0.000	False	LSU ribosomal protein L33p @ LSU ribosomal protein L33p, zinc-dependent
PGF_04457297	5.83	209	22	0.403	0.099	False	5-formyltetrahydrofolate cyclo-ligase (EC 6.3.3.2)
PGF_00016424	5.73	37	22	0.941	0.000	False	LSU ribosomal protein L36p @ LSU ribosomal protein L36p, zinc-dependent
PGF_00016395	5.60	60	22	0.724	0.020	False	LSU ribosomal protein L30p (L7e)
PGF_00016404	5.58	62	22	0.709	0.031	False	LSU ribosomal protein L32p @ LSU ribosomal protein L32p, zinc-dependent
PGF_00662997	5.44	266	22	0.334	0.073	False	Cobalamin synthase (EC 2.7.8.26)
PGF_10450086	5.31	165	22	0.414	0.161	False	Transcription termination protein NusB
PGF_10097367	5.31	834	22	0.184	0.623	False	Porphobilinogen deaminase (EC 2.5.1.61)
PGF_06632167	5.14	128	22	0.455	0.118	False	Signal recognition particle associated protein
PGF_08432396	4.99	445	22	0.237	0.522	False	Nicotinate-nucleotide adenylyltransferase (EC 2.7.7.18)

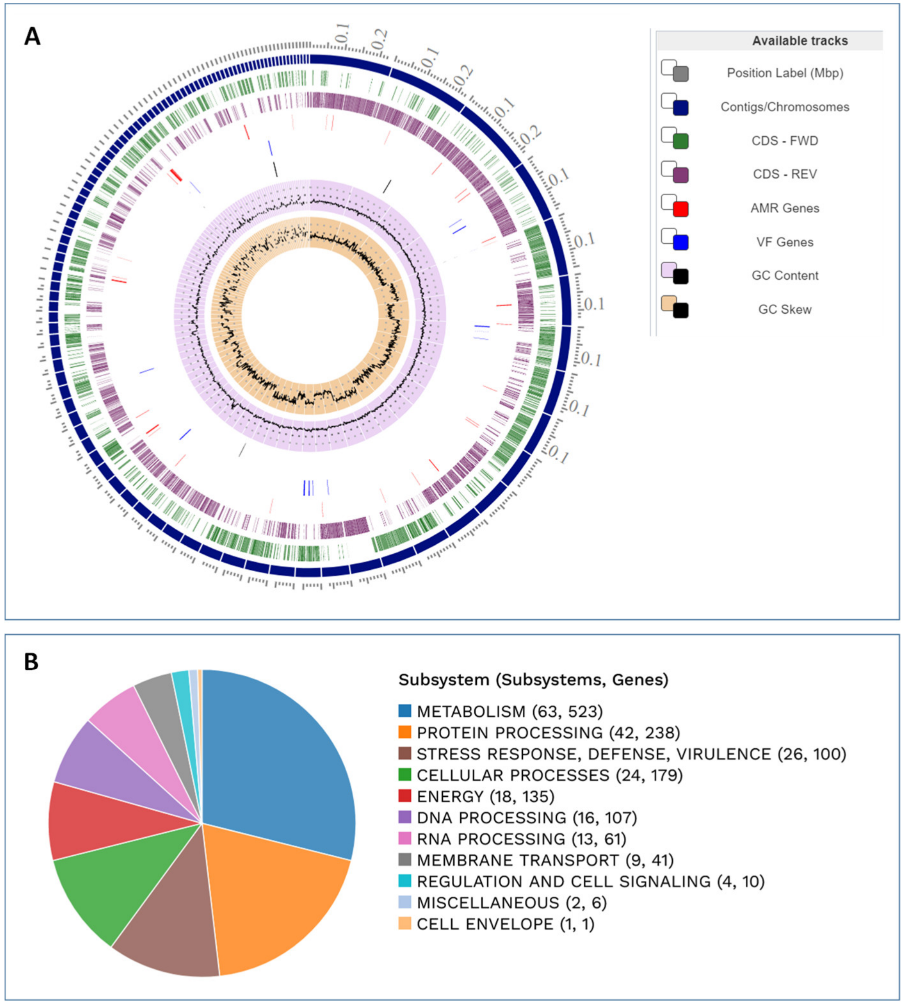

Figure S1: A: A circular graphical display of the distribution of the genome annotations in IRMC2505A. B: An overview of the subsystems of genes for the genome IRMC2505A.

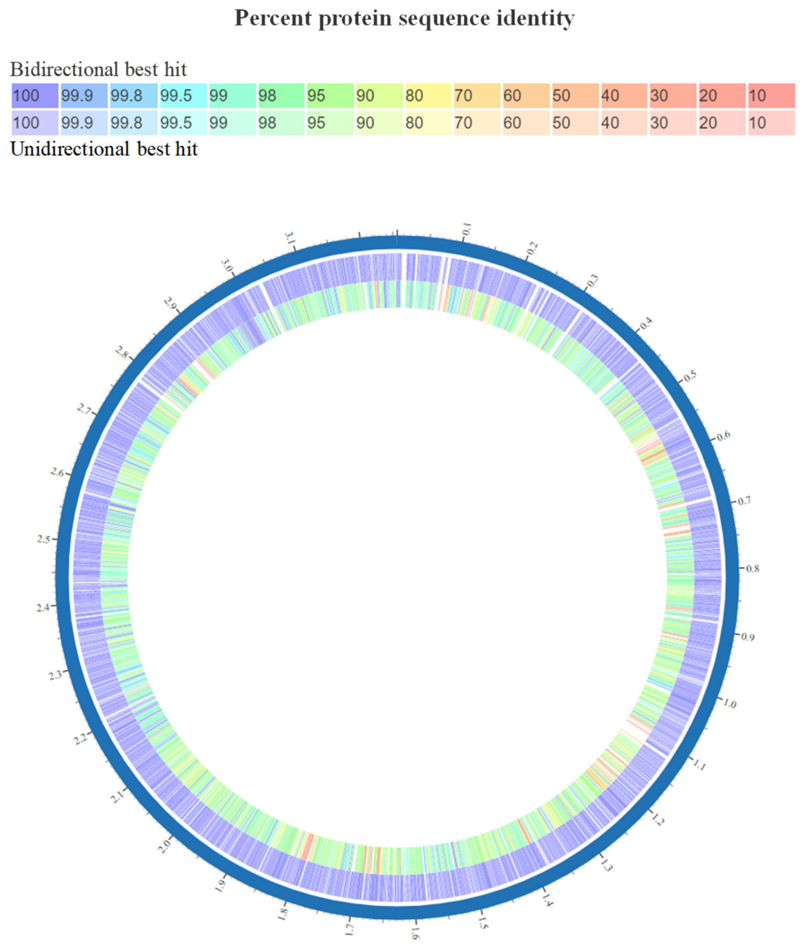

Figure S2: Proteome comparison. List of tracks, from outside to inside: *Clostridium perfringens* ATCC 13124 (195103.10) and *Clostridium perfringens* IRMC2505A (1502.2785).
